# Effect of Semaglutide on the Inflammatory Biomarker High-Sensitivity CRP in Patients With Established Cardiovascular Disease and Overweight or Obesity in SELECT: A Prespecified Secondary Analysis

**DOI:** 10.1161/CIRCULATIONAHA.125.074482

**Published:** 2026-08-18

**Authors:** Jorge Plutzky, Paweł Bogdański, Helen M. Colhoun, Selçuk Dağdelen, John E. Deanfield, Scott S. Emerson, G. Kees Hovingh, Steven E. Kahn, Kathrine Ekström, Gustavs Latkovskis, Michael Lehrke, Søren Hardt-Lindberg, Ildiko Lingvay, Stephen J. Nicholls, Tugce Kalayci Oral, Paula P. Terns, Søren Rasmussen, Paul M. Ridker, Donna H. Ryan, A. Michael Lincoff

**Affiliations:** 1 Division of Cardiovascular Medicine (J.P.), Brigham and Women’s Hospital, Harvard Medical School, Boston, MA.; 2 Center for Cardiovascular Disease Prevention and Cardiovascular Division (P.M.R.), Brigham and Women’s Hospital, Harvard Medical School, Boston, MA.; 3 Department of Treatment of Obesity, Metabolic Disorders, and Clinical Dietetics, Poznan University of Medical Sciences, Poland (P.B.).; 4 Institute of Genetics and Cancer, University of Edinburgh, Scotland (H.M.C.).; 5 Department of Endocrinology and Metabolism, School of Medicine, Hacettepe University, Ankara, Turkey (S.D.).; 6 Institute of Cardiovascular Science, University College London, UK (J.E.D.).; 7 Department of Biostatistics, University of Washington 7284 , Seattle (S.S.E.).; 8 Novo Nordisk A/S 509237 , Søborg, Denmark (G.K.H., K.E., S.H.-L., T.K.O., S.R.).; 9 Department of Vascular Medicine, Amsterdam University Medical Center, the Netherlands (G.K.H.).; 10 Department of Medicine, VA Puget Sound Health Care System and University of Washington, Seattle (S.E.K.).; 11 Faculty of Medicine and Life Sciences, University of Latvia, Riga (G.L.).; 12 Department of Internal Medicine I, University Hospital Aachen, RWTH Aachen University, Germany (M.L.).; 13 Department of Internal Medicine/Endocrinology and Peter O’Donnell Jr School of Public Health, UT Southwestern Medical Center, Dallas, TX (I.L.).; 14 Victorian Heart Institute, Monash University, Clayton, Victoria, Australia (S.J.N.).; 15 Dirección Médica, Cardiología Palermo–Centro de Investigaciones Clínicas, Buenos Aires, Argentina (P.P.T.).; 16 Pennington Biomedical Research Center, Baton Rouge, LA (D.H.R.).; 17 Department of Cardiovascular Medicine, Cleveland Clinic, Cleveland Clinic Lerner College of Medicine of Case Western Reserve University, OH (A.M.L.).

**Keywords:** atherosclerosis, cardiovascular diseases, glucagon-like peptide-1 receptor agonists, inflammation, obesity, semaglutide

## Abstract

**BACKGROUND:**

In SELECT (Semaglutide Effects on Heart Disease and Stroke in Patients With Overweight or Obesity), among 17 604 patients with known atherosclerotic cardiovascular disease and overweight or obesity, but not diabetes, randomization to the glucagon-like peptide-1 receptor agonist semaglutide significantly reduced the primary outcome of major adverse cardiovascular events (MACE; cardiovascular death, nonfatal myocardial infarction, or nonfatal stroke) compared with placebo (mean follow-up, 39.8 months). Inflammation, as indicated by plasma hsCRP (high-sensitivity C-reactive protein) level, is implicated as a biomarker predicting cardiovascular risk in obesity and atherosclerotic cardiovascular disease. SELECT provides a unique opportunity to study the relationship among hsCRP, obesity, weight loss, and MACE outcomes in semaglutide versus placebo groups.

**METHODS:**

In this prespecified SELECT substudy, we evaluated whether baseline hsCRP levels predicted MACE risk and examined the relationships between changes in hsCRP levels and time to first MACE, baseline body weight, weight loss, and other clinical measures among treatment groups over time (104, 208 weeks) using multiple approaches, including Cox modeling.

**RESULTS:**

Baseline hsCRP level, which was similar in the semaglutide (geometric mean 1.96 mg/L) and placebo (geometric mean 1.91 mg/L) groups, was prognostic of future MACE. The risk of MACE increased across baseline hsCRP level <2, 2–<10, and ≥10 mg/L subgroups, including significant associations with cardiovascular and all-cause death. Semaglutide reduced hsCRP levels (−37.8% [104 weeks]) and risk of MACE across all hsCRP subgroups. Greater reductions in ratio-to-baseline hsCRP with semaglutide were associated with greater weight loss, but preceded major weight loss, evident by 4 and 8 weeks, and occurred among those without weight loss. Semaglutide-associated changes in hsCRP were independent of low-density lipoprotein cholesterol levels, statin use, and atherosclerotic cardiovascular disease entry criteria. hsCRP reductions were found to be prognostic of decreased risk of MACE. Modeling suggests decreased inflammation as contributing in part to the benefits seen with semaglutide in SELECT.

**CONCLUSIONS:**

In SELECT, hsCRP data at baseline and in response to treatment with semaglutide support inflammation as a potential prognostic factor associated with cardiovascular risk in these generally well-treated patients with atherosclerotic cardiovascular disease and overweight or obesity but not diabetes. These findings suggest that the MACE reduction observed with semaglutide versus placebo in SELECT may have partially involved a decrease in inflammation.

**REGISTRATION:**

URL: https://www.clinicaltrials.gov; Unique identifier: NCT03574597.

**Clinical Perspective**

**What Is New?**

- In SELECT (Semaglutide Effects on Heart Disease and Stroke in Patients With Overweight or Obesity), a trial demonstrating that the glucagon-like peptide-1 receptor agonist semaglutide reduced future major adverse cardiovascular events in patients with overweight or obesity and atherosclerotic cardiovascular disease but not diabetes, baseline plasma hsCRP (high-sensitivity C-reactive protein) levels were prognostic of future major adverse cardiovascular events.
- Higher hsCRP levels (≥10 mg/L) were associated with a 2- to 4-fold higher risk of major adverse cardiovascular events, cardiovascular death, and all-cause death versus levels <2 mg/L.
- Semaglutide reduced hsCRP levels and cardiovascular risk across all baseline hsCRP levels, with effects preceding major weight loss, in those without weight loss, and independently of other measures (eg, cholesterol levels, statin use, atherosclerotic cardiovascular disease entry criteria).

**What Are the Clinical Implications?**

- These findings support the concept of inflammatory biomarkers such as hsCRP in identifying cardiovascular risk and add to the evidence that inflammation contributes to obesity-associated atherosclerotic cardiovascular disease and may be modifiable through glucagon-like peptide-1 receptor agonist treatment and weight loss.
- Modeling implicates the reduction in inflammation observed with semaglutide as contributing in part to the reduction in major adverse cardiovascular events reported in SELECT.
- Measuring hsCRP levels in patients with overweight or obesity and atherosclerotic cardiovascular disease may help clinicians identify ongoing cardiovascular risk and recognize patients who may benefit from weight loss intervention.

Inflammation promotes atherosclerosis and its complications, including myocardial infarction (MI), heart failure, and cardiovascular death.^1,2^ Circulating inflammatory biomarkers, including hsCRP (high-sensitivity C-reactive protein), predict future ischemic cardiovascular events independent of other cardiovascular risk factors, with assessment of inflammatory biomarkers now incorporated into management guidelines for atherosclerotic cardiovascular disease (ASCVD).^3,4^ Lowering low-density lipoprotein cholesterol (LDL-C) levels with statin therapy reduces hsCRP levels, which has been hypothesized as a potential contributor to the cardiovascular benefits of statins.^5^ In CANTOS (Canakinumab Anti-Inflammatory Thrombosis Outcome Study), the IL-1β (interleukin-1β) neutralizing antibody canakinumab reduced major adverse cardiovascular events (MACE; nonfatal MI, nonfatal stroke, and cardiovascular death) without altering LDL-C levels in patients with hsCRP ≥2 mg/L, indicating that a decrease in inflammation may mitigate ASCVD risk.^6^ The anti-inflammatory agent colchicine has been reported to decrease the risk of MACE and is approved for ASCVD risk reduction,^7^ but did not reduce the primary composite cardiovascular end point compared with placebo among patients with a recent MI,^8^ highlighting the need for additional data regarding hsCRP and inflammation in ASCVD in additional patient populations and clinical trials.

Inflammation has also been linked to overweight and obesity.^9^ Inflammatory biomarkers, including hsCRP level, are associated directly with adipose tissue volume, visceral and pericardial fat mass, waist circumference, and body mass index (BMI).^10–12^ Inflammatory cells, including T cells and macrophages, are present in adipose tissue, with these and other cells, including adipocytes themselves, releasing inflammatory modulators with potential local and systemic effects.^13^ In line with the connection between excess adiposity and systemic inflammation, body weight loss is associated with a decrease in inflammatory signals and biomarkers.^14–16^

Glucagon-like peptide-1 receptor agonists (GLP-1 RAs) induce substantial weight loss and are known to reduce MACE in patients with type 2 diabetes.^17,18^ The effects of the GLP-1 RA semaglutide on reducing body weight and MACE in type 2 diabetes are associated with decreased inflammatory biomarkers, including hsCRP levels, as reported in weight loss–focused semaglutide trials.^19^ Recently, in SELECT (Semaglutide Effects on Heart Disease and Stroke in Patients With Overweight or Obesity; URL: https://www.clinicaltrials.gov; Unique identifier: NCT03574597)—a multicenter, double-blind, randomized controlled trial performed in 17 604 patients with established ASCVD and overweight or obesity but not diabetes—once-weekly semaglutide 2.4 mg was found to reduce MACE by 20% compared with placebo.^20^ Given the evidence that hsCRP levels predict the risk of MACE and the established effects of GLP-1 RAs on multiple cardiovascular risk factors, this prespecified SELECT substudy was designed to test the hypotheses that baseline hsCRP levels would predict the risk of future MACE independent of statin use and LDL-C levels and that hsCRP lowering with semaglutide versus placebo would be associated with a decreased risk of MACE. The analyses of the SELECT data undertaken here provide a unique opportunity to examine the relationship between inflammatory biomarker hsCRP levels in these patients at entry and in response to treatment with semaglutide versus placebo, as well as the SELECT cohort’s baseline characteristics, changes in body weight on trial, and cardiovascular outcomes.

## Methods

Authorized researchers can request access to clinical study data by submitting a research proposal for review and approval by Novo Nordisk and an internal independent review panel. If the research supports a regulatory application, requests will be considered after the product and its intended use are approved in both the European Union and the United States. Participants’ clinical data will be anonymized, following an approved internal process, before data are shared to external third parties. Details on requesting access to clinical data are available at novonordisk-trials.com.

### Trial Design

In brief, the phase 3 SELECT trial was a randomized, double-blind, placebo-controlled, event-driven trial conducted at 804 sites in 41 countries. Patients were enrolled between October 2018 and March 2021.^20–22^ SELECT compared once-weekly semaglutide dose-escalated to 2.4 mg with placebo when added to standard of care for prevention of MACE in patients with established ASCVD and overweight or obesity, without diabetes. The SELECT protocol was approved by the institutional review board and ethics committee at each participating center. All patients provided written informed consent before commencement of any trial-specific activity. The first author had full access to all the data and vouches for the accuracy and integrity of the data and the statistical analyses. This report adhered to the CONSORT (Consolidated Standards of Reporting Trials) 2025 reporting guidelines (available in the Supplemental Material).

### Patients

Eligible patients were ≥45 years of age with a BMI ≥27 kg/m^2^ and established ASCVD, defined as ≥1 of the following: previous MI, previous ischemic or hemorrhagic stroke, or symptomatic peripheral artery disease. Exclusion criteria included HbA_1c_ (glycated hemoglobin) ≥48 mmol/mol (≥6.5%); history of type 1 or 2 diabetes; end-stage kidney disease; previous MI, stroke, hospitalization for unstable angina pectoris, or transient ischemic attack within 60 days of screening; or New York Heart Association class IV heart failure. Patients were randomized (1:1) to escalating dosages of once-weekly subcutaneous semaglutide over 16 weeks to a target dose of 2.4 mg or placebo.^20,22^ Patients were followed for a duration of 39.8±9.4 months (mean±SD) for incident cardiovascular events.

### Baseline Characteristics and Outcomes

**Figure 1.**
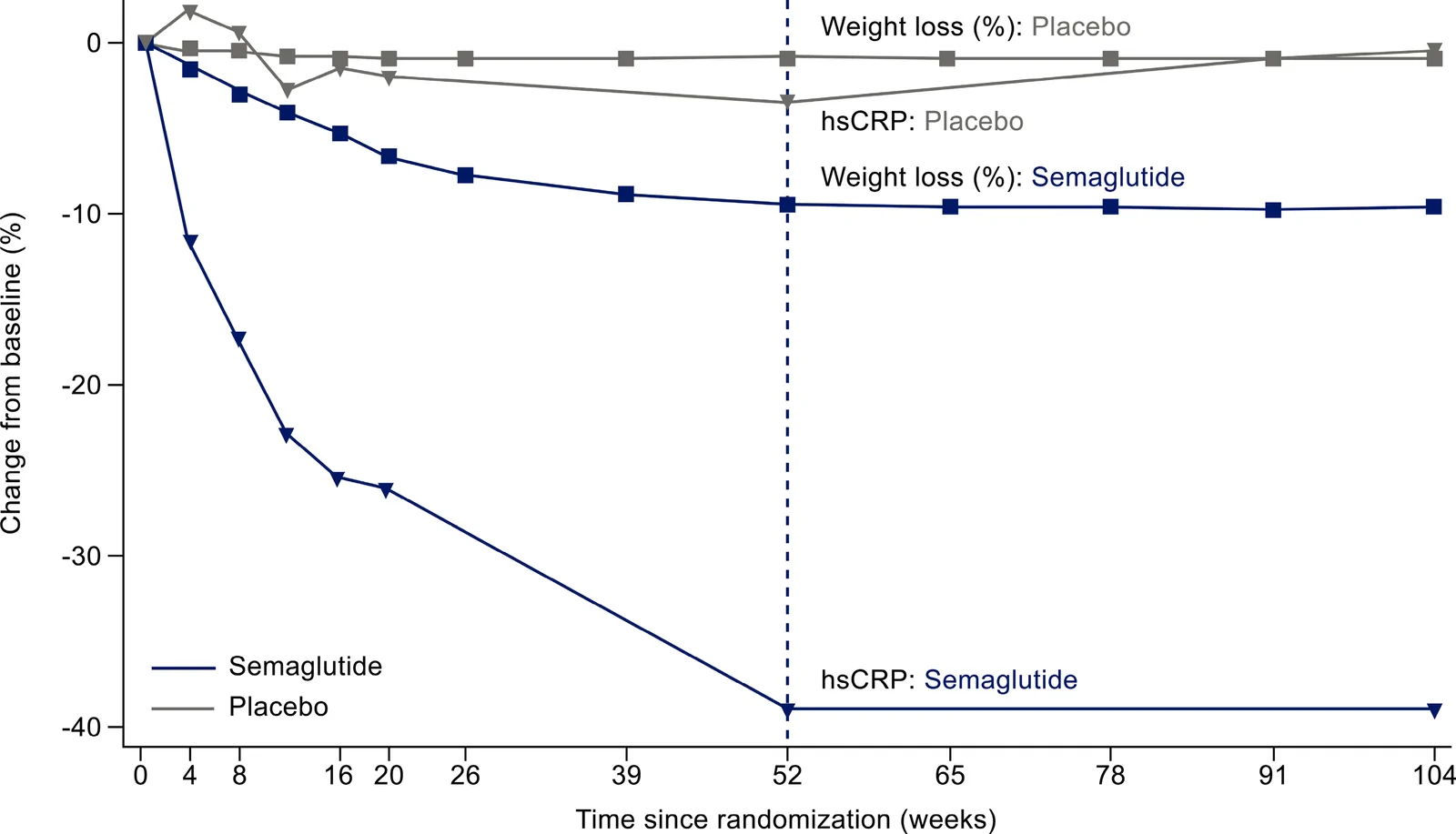
**High-sensitivity CRP and body weight percentage change from baseline to week 104.** hsCRP data are observed geometric mean (SE) ratios to baseline and shown as percentage changes using the formula −1*(1−geometric mean ratio)*100% using data from the in-trial observation period. Measurements at weeks 4 through ‍16 were for a subpopulation of 6011 randomized patients from European countries in whom extra laboratory measurements were conducted due to mandated safety requirements. Baseline hsCRP values for the entire cohort were 1.87 mg/L (median) and 1.96 mg/L (geometric mean) for semaglutide and 1.80 mg/L (median) and 1.91 mg/L (geometric mean) for placebo. Weight data (mean [SE]) are observed percentage change from baseline in the in-trial period. Baseline body weights were 96.5 kg (mean) and 96.8 kg (mean) for semaglutide and placebo, respectively. In linear continuous modeling among placebo patients, each 25% lower hsCRP level at baseline was associated with 6% lower risk of MACE (HR, 0.94 [95% CI, 0.93, 0.96]); each 5 kg lower weight at baseline (≈5.1% of the mean weight of 96.8 kg) was associated with 1% lower risk of MACE (HR, 0.99 [95% CI, 0.97, 1.01]). HR indicates hazard ratio; hsCRP, high-sensitivity C-reactive protein; and MACE, major adverse cardiovascular events.

The primary outcome of SELECT was MACE, comprising death from cardiovascular causes, nonfatal MI, or nonfatal stroke. MACE outcomes have been reported previously.^20^ The current secondary analyses examine the interrelationships among prespecified measures of inflammation, body weight measures, and cardiovascular events in both treatment arms during SELECT, including baseline hsCRP and subsequent MACE outcomes. Regarding changes during the trial, analyses primarily consider proportionate change in hsCRP level at 104 weeks after randomization (ratio of hsCRP measurements at week 104 to the baseline measurement for each patient), the percent change in body weight from baseline to week 104, and the time from randomization to each of 3 events (MACE, death from cardiovascular cause, all-cause death). Descriptive analyses consider the time course of hsCRP and body weight over all protocol-specified measurements. Blood samples were collected at patient enrollment or randomization, then subsequently at weeks 20, 52, 104, 156, and 208, and at the end of the patient’s treatment phase. hsCRP assessment was subsequently performed by a high-sensitivity assay in a central laboratory. Among patients from certain European countries, additional blood tests were undertaken between weeks 4 and 16, given mandated safety requirements (Austria, Belgium, Czech Republic, Denmark, Finland, France, Germany, Greece, Hungary, Republic of Ireland, Italy, Latvia, the Netherlands, Norway, Poland, Portugal, Romania, Spain, Sweden, and the United Kingdom; Figure 1).

### Statistical Analysis

An overview of statistical analyses and approaches is provided here; additional detail is provided in the Methods Overview in the Supplemental Material, including alternative analyses that explored sensitivity to the modeling assumptions used in detecting trends as well as the handling of missing data. Analyses followed the intention-to-treat principle and were performed on the full analysis set, defined as all unique randomized patients grouped according to the treatment assigned at randomization and based on the in-trial period: the time from randomization to the end-of-trial visit, death, withdrawal of consent, or last trial site contact, whichever occurred first. Baseline characteristics included sex, hsCRP level, LDL-C level, cardiovascular inclusion criteria, statin use, BMI, HbA_1c_, urine albumin-to-creatine ratio, age, region, race, ethnicity, heart failure, estimated glomerular filtration rate, and KDIGO (Kidney Disease: Improving Global Outcomes) category, as considered across baseline hsCRP subgroups.

The relationship between changes in hsCRP levels from baseline to week 104 and observed weight loss categories over the same period were analyzed using a mixed model for repeated measurements, with weight loss categories by treatment group as a fixed factor and baseline value as a covariate, all nested within visit. hsCRP values were log-transformed before analyses. Analyses were performed using 2 different weight loss categorizations based on observed change in body weight from randomization to week 104: subgroups of body weight reduction of <5%, 5%–<10%, 10%–<15%, and ≥15%; and body weight reduction of <2% or >2%. Body weight analyses used in-trial data, whereas the sensitivity analysis used first on-treatment data. Subgroup analyses of the ratios at week 104 to baseline hsCRP were studied using an ANCOVA with treatment group, subgroup, and treatment group-by-subgroup interaction as fixed factors, and baseline value as a covariate. Multiple imputation (n=500) through linear regression was used to impute missing week 104 data separately for each treatment group. This prespecified imputation model included the baseline value as a covariate and was fitted to all patients with a measurement regardless of treatment status at week 104. Missing data were defined as observable data that had been planned to be collected but not present in the database. Hence, data that are absent from the database due to death or administrative censoring were not considered missing and not imputed. A Markov Chain Monte Carlo method was used to impute data points to achieve monotone data missingness patterns for each patient. Sensitivity analyses were performed using the first on-treatment period, defined as the on-treatment observation period until the first time being off treatment for 5 consecutive weeks (35 days). Sensitivity analyses also evaluated the imputation scheme using last observation carried forward for patients who died during the trial and baseline and observed postbaseline values to impute for those patients who were alive during the trial, segregated by treatment arm, and analyzed using linear regression. Additional background information and rationale for the use of last observation carried forward, including handling of data from patients who died during the trial, are provided in the Supplemental Material.

Hazard ratios (HRs) for the subgroup analyses of selected cardiovascular outcomes (MACE, cardiovascular-related death, all-cause death) comparing semaglutide versus placebo were estimated using a Cox proportional hazards model with treatment group, subgroup, and treatment group-by-subgroup interactions as fixed factors. Data from patients who withdrew from the trial, died from causes not included in the end point, or were lost to follow-up were censored at the time of withdrawal, death, or last contact with the investigator. Cumulative incidence plots by subgroup and treatment used the Aalen–Johansen estimator.^23^ HRs across hsCRP subgroups were estimated using a Cox proportional hazard model with the hsCRP subgroup of <2 mg/L as reference and adjusted for treatment group.

Analyses of outcomes accounting for time-dependent hsCRP changes during the trial were based on a Cox proportional hazards model with treatment group as a fixed factor; baseline LDL-C level, statin use, and baseline log hsCRP level as covariates; and log ratio-to-baseline hsCRP as a time-dependent covariate. Observed hsCRP values were carried forward each day until a new value was obtained at a subsequent trial visit or otherwise left unchanged. A sensitivity analysis of the selected cardiovascular outcomes was performed using the same model but using only data from the first on-treatment period. Significance levels were set at 5% (2-sided), with no adjustment for multiplicity. SAS version 9.4 was used for the analyses.

Given observed differences between male and female patients with respect to the treatment effect on proportionate changes in hsCRP level at week 104, post hoc analyses are presented in the Supplemental Material that consider the primary analyses within each subgroup defined by sex, as noted where relevant in the Results.

## Results

### SELECT Patient Characteristics According to Baseline hsCRP Level

The geometric mean baseline hsCRP level was 1.96 mg/L (interquartile range, 0.89–4.18; n=8732) for patients assigned to semaglutide and 1.91 mg/L (interquartile range, 0.86–4.06; n=8753) for those in the placebo arm. Baseline hsCRP levels were <2 mg/L (n=9239), 2–<10 mg/L (n=6913), or ≥10 mg/L (n=1333; Table). The mean (SD) age was similar across treatment groups (60.9±8.8 to 61.0±8.8 years) and >50% of patients were men across all hsCRP categories. Body weight, BMI, and waist circumference were higher in patients with increasing baseline hsCRP cut points of <2 mg/L, 2–<10 mg/L, and ≥10 mg/L (Table). hsCRP levels were higher in women and Black patients and lower in Asian patients. Patients with higher baseline hsCRP levels tended to have a greater burden of ASCVD, with ≥2 ASCVD entry criteria, or a history of stroke compared with patients with lower baseline hsCRP values. Similar trends were evident when grouping patients by hsCRP tertiles (Table S1).

**Table.**
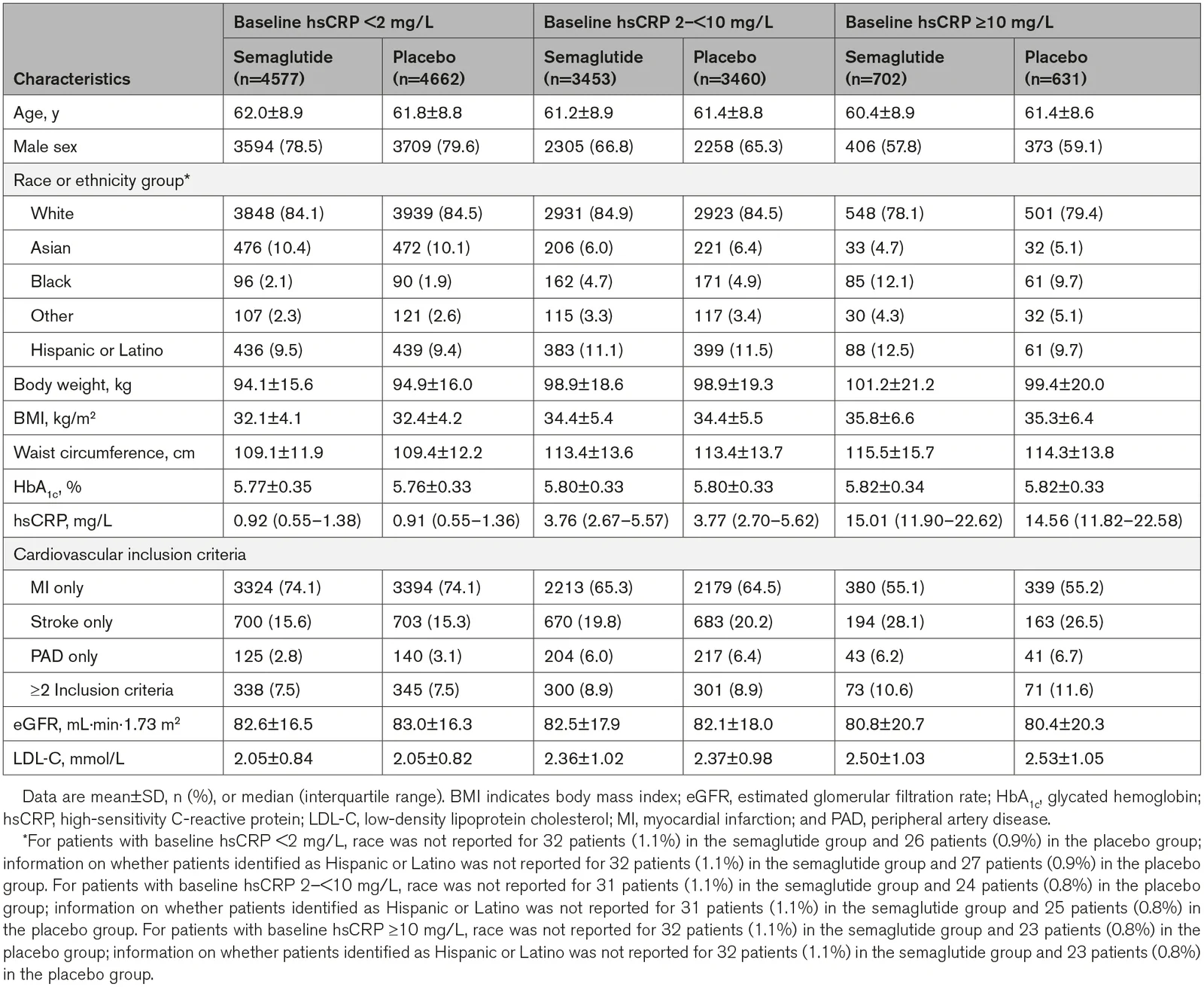
Baseline Characteristics by Baseline High-Sensitivity CRP Levels

### Change in hsCRP Level Over Time in SELECT

The semaglutide group had a significant reduction in geometric mean hsCRP level (−37.8% [95% CI, −39.7, −35.9], placebo-corrected) over 104 weeks of the study (Figure 1). Very similar hsCRP reductions are seen using other imputation schemes for missing data and patients who died during the trial (hsCRP −37.6% [95% CI, −39.4, −35.7]; Methods Overview in the Supplemental Material). Among those assigned to semaglutide, the ratio-to-baseline hsCRP declined rapidly, reaching a nadir (0.62) at the 52-week measurement and remaining at this level when assayed at 104 weeks of treatment. The temporal patterns of the semaglutide treatment arm approaching nadir hsCRP and nadir body weight at 52 weeks are similar (Figure 1). Notably, among patients with early hsCRP measurements (<12 weeks) during the trial, the ratio-to-baseline hsCRP decreased in the semaglutide group by 12% by 4 weeks and 19% by 8 weeks; at these same time points, which preceded full semaglutide dose escalation (0.5 mg/week at 8 weeks), semaglutide-induced body weight loss was 1.4% and 2.8%, respectively. In comparison, in the placebo group at 4 and 8 weeks, the ratio-to-baseline hsCRP decreased by 1.8% and increased by 0.1%, and body weight was reduced by 0.3% and 0.4%, respectively. The temporal patterns of hsCRP levels and body weight changes were robust when alternative imputation strategies were used and extended to 208-week measurements (Figure S1 and Table S2). Absolute changes in hsCRP levels to week 104 were similar (Figure S2).

### Change in hsCRP Level According to Baseline SELECT Patient Characteristics

**Figure 2.**
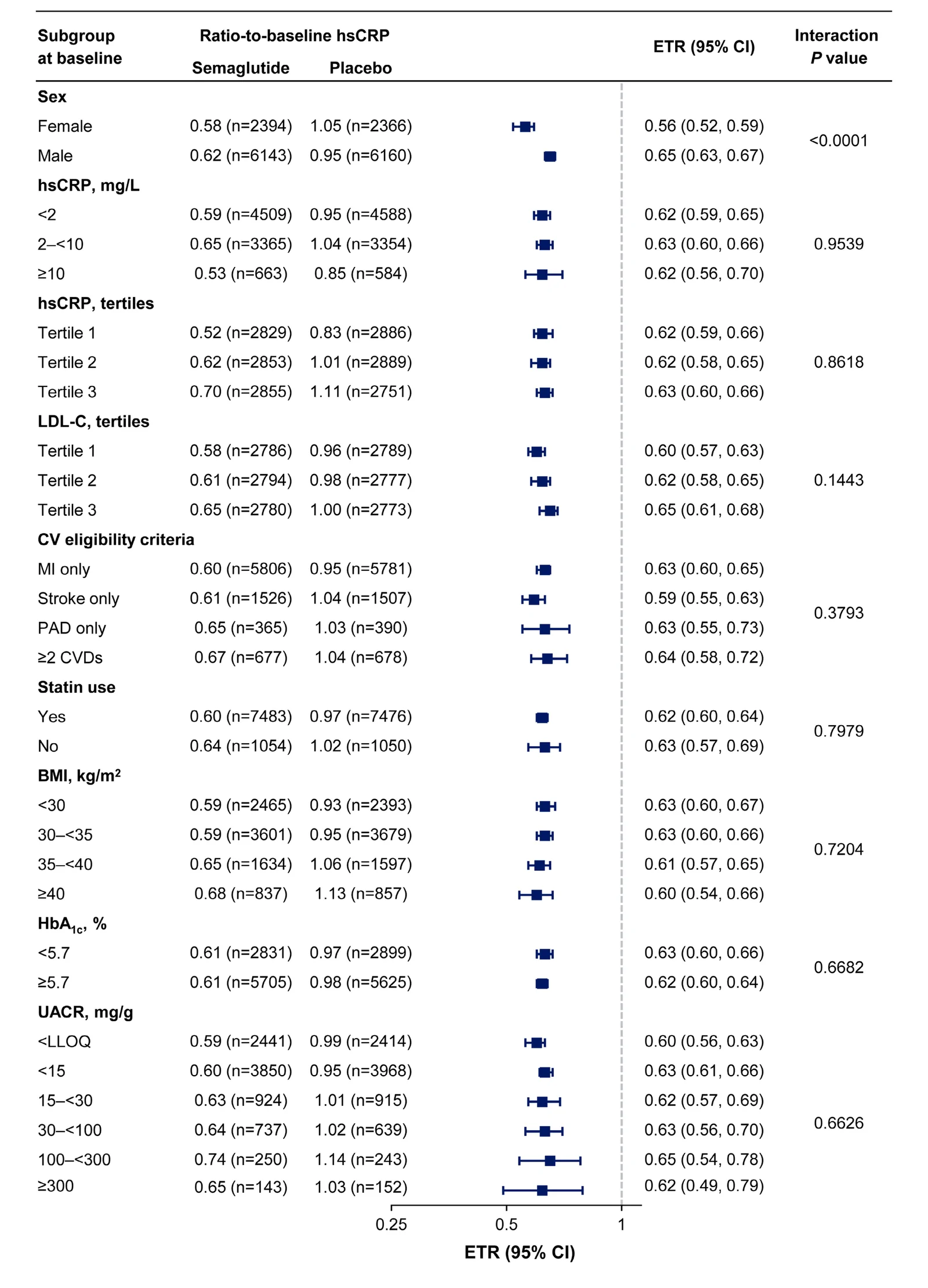
**Forest plot of ratio-to-baseline high-sensitivity CRP levels at week 104 by selected baseline subgroups and treatment arm.** Data are estimated geometric mean ratios to baseline and ETRs, analyzed using an ANCOVA with treatment group, subgroup, and treatment group-by-subgroup interaction as fixed factors, and baseline value as a covariate, using in-trial data from the observation period. Multiple imputation through linear regression was used to impute missing week 104 data separately for each treatment group. The model included the baseline value as a covariate and was fitted to all patients with a measurement regardless of treatment status at week 104. Mean estimates were adjusted according to observed baseline distribution. The hsCRP observations were log-transformed before analysis and the estimates back-transformed afterwards. n denotes the number of patients contributing to the analysis. BMI indicates body mass index; CV, cardiovascular; CVD, cardiovascular disease; ETR, estimated treatment ratio; HbA_1c_, glycated hemoglobin; hsCRP, high-sensitivity C-reactive protein; LDL-C, low-density lipoprotein cholesterol; LLOQ, lower limit of quantification; MI, myocardial infarction; PAD, peripheral artery disease; and UACR, urine albumin-to-creatinine ratio.

The changes in ratio-to-baseline hsCRP at week 104 across patient subgroups and demographics between treatment arms are shown in Figure 2. No effect modification was observed on the ratio-to-baseline hsCRP at week 104 with semaglutide versus placebo when analyzed by baseline hsCRP cut points (<2 mg/L, 2–<10 mg/L, ≥10 mg/L), tertiles of hsCRP, LDL-C level, statin use, cardiovascular disease eligibility criteria, BMI, baseline HbA_1c_ (<5.7%, ≥5.7%), or urine albumin-to-creatine ratio groups. Women had a greater decrease in ratio-to-baseline hsCRP than men (estimated treatment ratio [ETR; 95% CI]; women, 0.56 [0.52, 0.59], men, 0.65 [0.63, 0.67]; *P*<0.0001; Figure 2) and a similar trend was observed in younger versus older patients (Table S3). Analysis in more precisely defined baseline hsCRP categories of <1 mg/L versus 1–<2 mg/L versus 2–<5 mg/L versus 5–<10 mg/L versus ≥10 mg/L showed that the effect of semaglutide on ratio-to-baseline hsCRP versus placebo was significant and homogeneous irrespective of baseline hsCRP level (Figure S3). In linear continuous modeling, baseline hsCRP level was strongly prognostic of proportionate change in hsCRP level in a linear manner, with similar slopes in both treatment arms, with similar effects in men and women (Table S4).

### Baseline hsCRP Level and Cardiovascular Outcomes in SELECT

**Figure 3.**
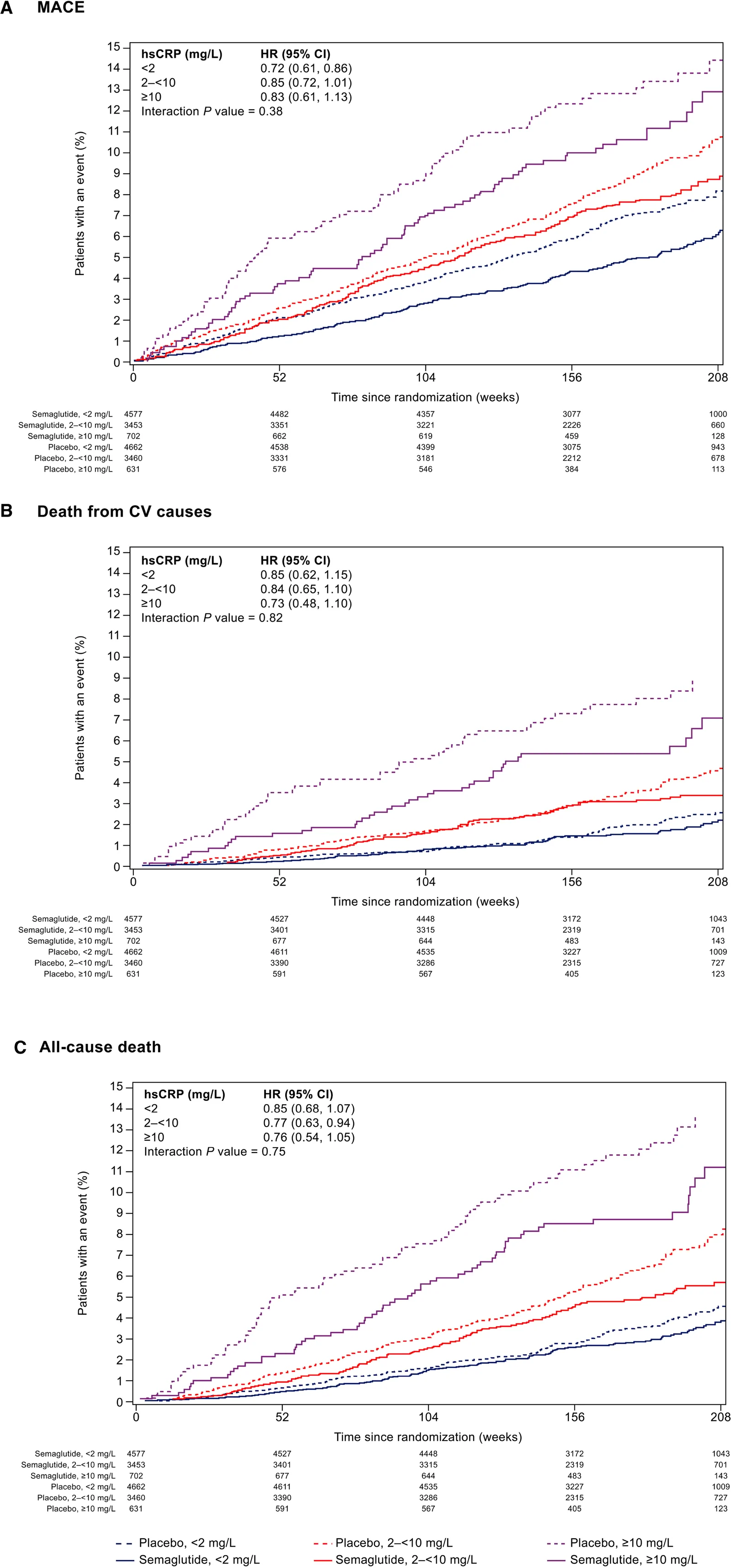
**Cumulative incidences of time to major adverse cardiovascular event, death from cardiovascular causes, and all-cause death by baseline high-sensitivity CRP groups and treatment arm.** Cumulative incidences of time to (**A**) MACE, (**B**) death from CV causes, and (**C**) all-cause death by baseline hsCRP groups (<2, 2–<10, ≥10 mg/L) and treatment arm. Data are the observed (ie, as-measured) cumulative incidence rates of patients experiencing their first occurrence of each CV outcome, calculated using the Aalen–Johansen estimator accounting for non–CV-related death as a competing risk, and the estimated HRs, analyzed using a Cox proportional hazards model with treatment group, subgroup, and treatment group-by-subgroup interactions as fixed factors, using in-trial data from the observation period. Numbers underneath panels represent the number of patients at risk. CV indicates cardiovascular; HR, hazard ratio; hsCRP, high-sensitivity C-reactive protein; and MACE, major adverse cardiovascular events.

**Figure 4.**
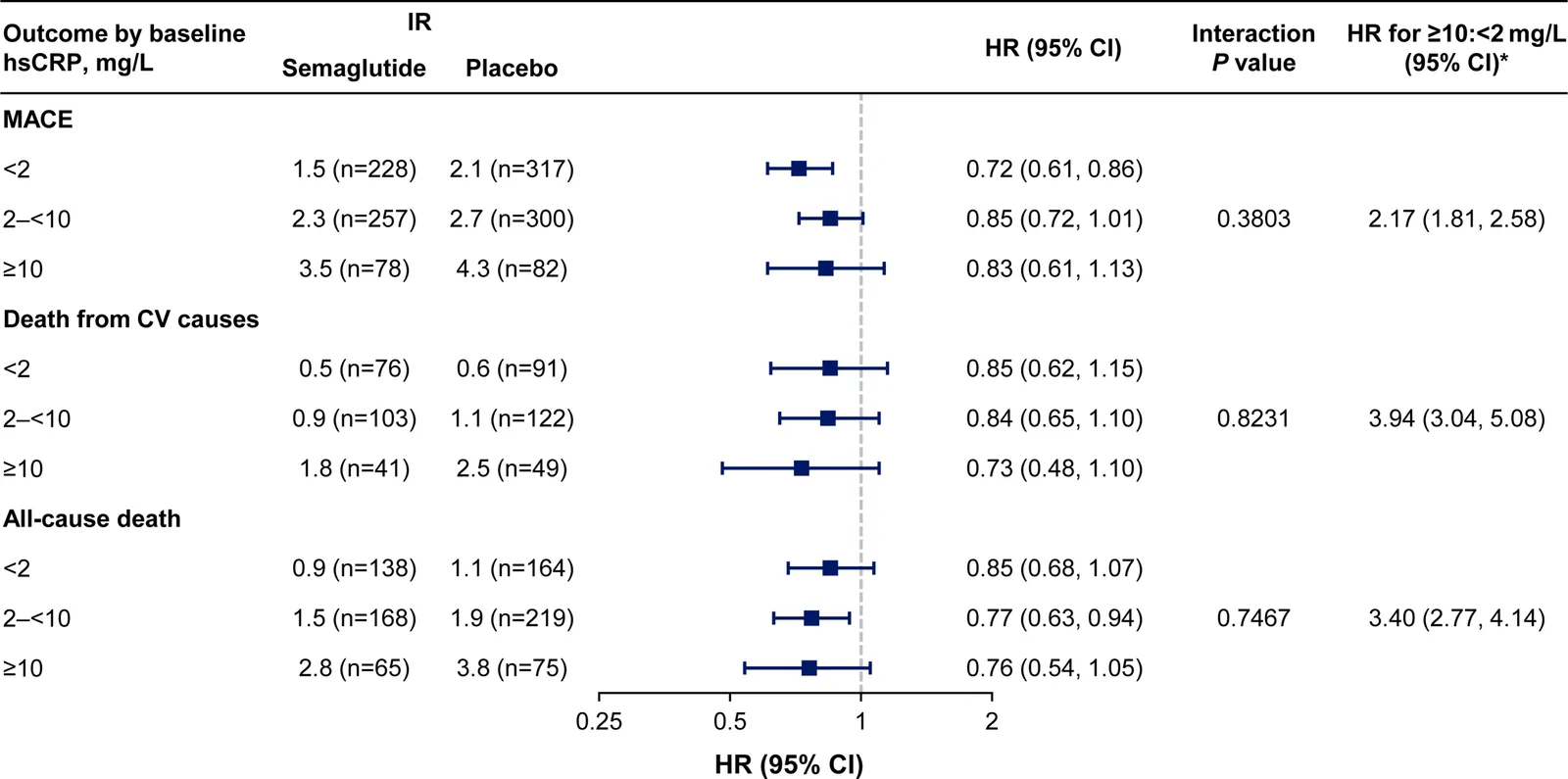
**Hazard ratios for semaglutide versus placebo for major adverse cardiovascular events, death from cardiovascular causes, and all-cause death according to baseline high-sensitivity CRP levels.** Forest plot showing the HRs for semaglutide versus placebo for MACE, death from CV causes, and all-cause death according to baseline hsCRP (<2, 2–<10, ≥10 mg/L) and relative to hsCRP <2 mg/L. Data are the estimated HRs, analyzed using a Cox proportional hazards model with treatment group, subgroup, and treatment group-by-subgroup interactions as fixed factors, using in-trial data from the observation period. n denotes the number of patients who had an event. IR indicates number of first events per 100 years of observation time. *Ratio of the hazard in the subgroup with the highest baseline values (≥10 mg/L) relative to the hazard in the subgroup with <2 mg/L estimated from a Cox proportional hazards model with subgroup as a fixed factor (with <2 mg/L as the reference) adjusted for treatment group. CV indicates cardiovascular; HR, hazard ratio; hsCRP, high-sensitivity C-reactive protein; IR, incidence rate; and MACE, major adverse cardiovascular events.

Higher baseline hsCRP level among SELECT patients was associated with a higher incidence of MACE (Figure 3A), death from cardiovascular causes (Figure 3B), and all-cause death (Figure 3C). For all outcomes across all baseline hsCRP levels, random allocation to semaglutide compared with placebo resulted in similar magnitudes of event reduction (all *P*_interaction_>0.05). Similar results are seen when dividing the study population by hsCRP tertiles rather than by clinical hsCRP cut points (Figure S4). A forest plot of MACE, death from cardiovascular causes, and all-cause death according to baseline hsCRP groups (<2 mg/L, 2–<10 mg/L, ≥10 mg/L) is provided in Figure 4. The HR (95% CI) for MACE, death from cardiovascular causes, and all-cause death between patients with hsCRP ≥10 mg/L versus <2 mg/L was 2.17 (1.81, 2.58), 3.94 (3.04, 5.08), and 3.40 (2.77, 4.14), respectively (Figure 4), suggesting a 2- to 4-fold higher risk of these outcomes for those with hsCRP ≥10 mg/L compared with patients with hsCRP <2 mg/L across treatment groups. In linear continuous modeling, lower baseline hsCRP level was found to be strongly prognostic of lower risk of MACE in a linear manner, with similar slopes in both the semaglutide arm (HR, 0.92 per 25% lower baseline hsCRP [95% CI, 0.91, 0.94]) and placebo arm (HR, 0.94 per 25% lower baseline hsCRP [95% CI, 0.93, 0.96]), as observed in both men and women (Table S5). Using similar modeling of baseline weight, a significant linear trend toward lower risk of MACE is seen only with lower baseline weight in the semaglutide group (HR, 0.96 per 5 kg lower baseline weight [95% CI, 0.94, 0.99]) but not in the placebo arm, again with similar such trends in post hoc analyses of men and women (Table S5).

### Change in hsCRP Level According to Weight Change During SELECT

**Figure 5.**
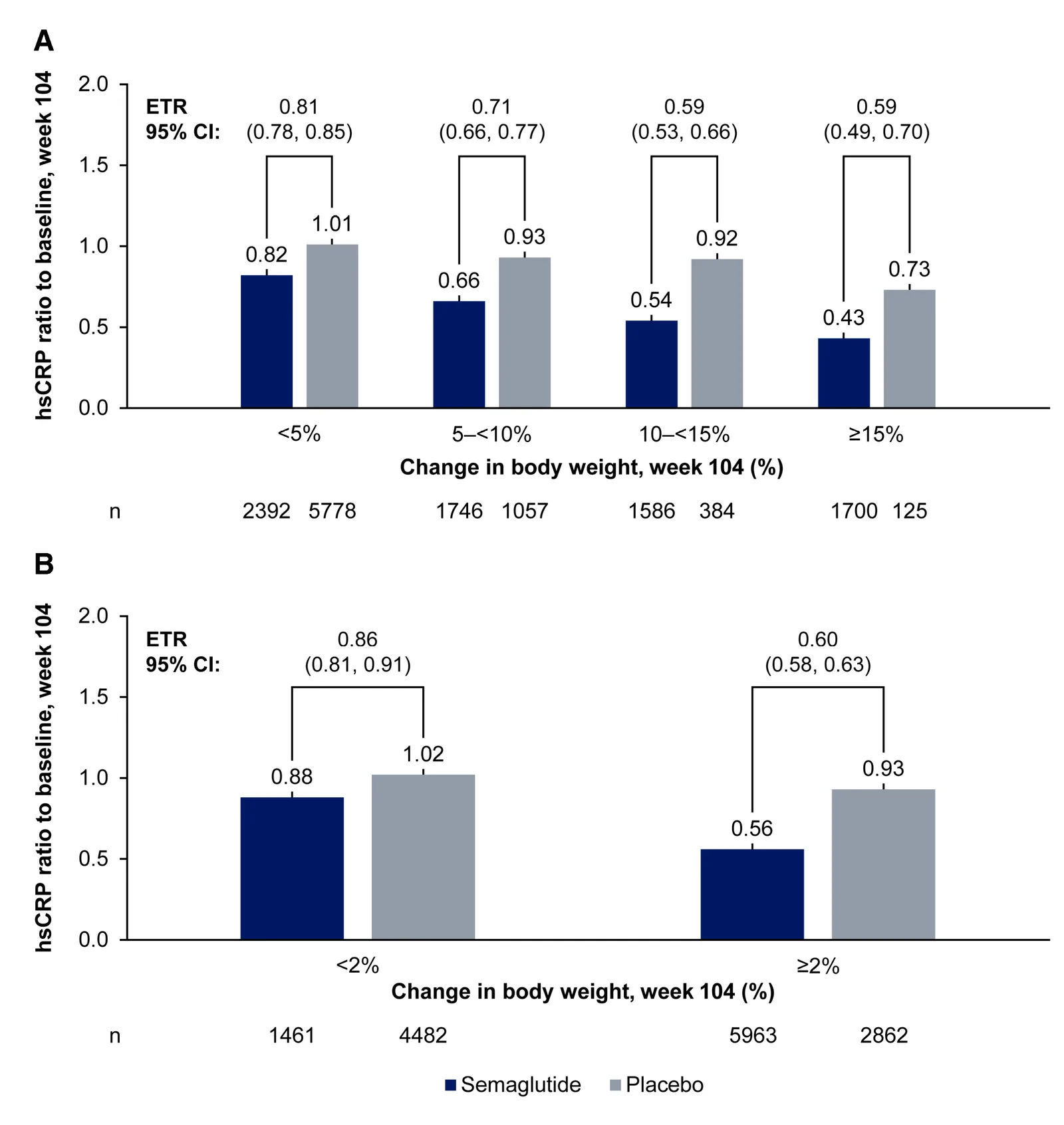
**Ratio-to-baseline high-sensitivity CRP at week 104 by observed groups of body weight change at week 104. A**, Ratio-to-baseline hsCRP according to changes in body weight in groups of <5%, 5%–<10%, 10%–<15%, and ≥15%. **B**, Ratio-to-baseline hsCRP in body weight groups of <2% and ≥2%. For both **A** and **B**, data are estimated geometric mean ratios to baseline and ETRs, analyzed using a mixed model with treatment group, subgroup, and treatment group-by-subgroup interaction as fixed factors, and baseline body weight and hsCRP as covariates, using observed (ie, as-measured) in-trial data from the observation period. The model included the baseline value as a covariate and was fitted to all patients with a measurement regardless of treatment status at week 104. Mean estimates were adjusted according to observed baseline distribution. Baseline hsCRP was log-transformed before analysis and the estimates back-transformed afterward. n denotes the number of patients contributing to the analysis. *P*_interaction_<0.001. Linear trend test *P*<0.001. ETR indicates estimated treatment ratio; and hsCRP, high-sensitivity C-reactive protein.

The ratio-to-baseline hsCRP level within different weight loss categories was analyzed during the trial, comparing semaglutide versus placebo treatment groups (Figure 5). A larger decrease in ratio-to-baseline hsCRP with greater weight loss was seen among those randomized to semaglutide, with geometric means of proportionate change in hsCRP level of 0.82, 0.66, 0.54, and 0.43 across weight loss categories of <5%, 5%–<10%, 10%–<15%, and ≥15%, respectively (Figure 5A). For patients receiving placebo, ratio-to-baseline hsCRP remained mostly unchanged except for those patients experiencing weight loss of ≥15% (1.01, 0.93, 0.92, and 0.73, respectively). As shown, relatively few placebo-treated patients lost 10%–<15% (n=384) or ≥15% (n=125) of body weight. In comparisons across these nonrandomized subgroups, the effect of semaglutide on hsCRP levels was larger compared with placebo in patients with greater weight loss (ETR [95% CI], 0.81 [0.78, 0.85], 0.71 [0.66, 0.77], 0.59 [0.53, 0.66], and 0.59 [0.49, 0.70], respectively [*P*_interaction_<0.001]).

Changes in weight and hsCRP level in SELECT were analyzed to further consider correlations in how these 2 measures changed over time, comparing week 104 with week 208 measurements, and in respect to one another (Table S6). In the placebo group, the correlations between week 104 and week 208 measurements were moderately high for both percent change in body weight from baseline and the ratio of postrandomization hsCRP level to baseline hsCRP measurements (0.627 and 0.536, respectively). However, in the placebo arm, the measures of change in body weight and hsCRP level were not highly correlated to each other (0.080 at week 104, 0.062 at week 208). In contrast, among semaglutide-treated patients, all measurements were significantly more highly correlated with each other than seen in the placebo arm (*P*<0.0001). A higher correlation between the percent change in body weight from baseline and the ratio of postrandomization hsCRP level to baseline is present in semaglutide-treated patients (0.266 at week 104, 0.250 at week 208). Similar results are obtained in on-treatment analyses at week 104 (Figure S5). In linear continuous modeling using alternative imputation of missing data (Table S7A), in the semaglutide arm, greater weight loss by week 104 was found to be strongly prognostic of lower hsCRP level in a linear manner, with significantly steeper slopes (*P*_interaction_<0.001; HR, 0.87 per 5% decrease in body weight [95% CI, 0.86, 0.88]) than those seen in the placebo arm (HR, 0.94 per 5% decrease in body weight [95% CI, 0.93, 0.96]). Among patients with weight gain, those receiving semaglutide (n=1212) had greater reductions in ratio-to-baseline hsCRP (geometric mean, 0.85 [95% CI, 0.80, 0.90]) than patients on placebo who gained weight (n=3984; geometric mean, 1.06 [95% CI, 1.02, 1.09]; Table S7A). Similar overall associations between proportionate change in hsCRP level and percentage change in weight were observed in similar post hoc analyses among male and female subgroups (Table S7B and S7C).

Whereas semaglutide-treated patients with at least some weight loss (≥2% body weight; n=5963) had greater reductions in ratio-to-baseline hsCRP (0.56) than those on placebo in a similar weight loss group (n=2862; 0.93; ETR, 0.60 [95% CI, 0.58, 0.63]), among patients without significant weight loss (<2% body weight), a decrease in ratio-to-baseline hsCRP was seen in those receiving semaglutide (n=1461; 0.88) but not placebo (n=4482; 1.02; ETR, 0.86 [95% CI, 0.81, 0.91]; *P*_interaction_<0.001; Figure 5B). Similar patterns are seen in on-treatment analyses of those with nearly identical ETRs as shown previously. The decrease in ratio-to-baseline hsCRP among the semaglutide group who did not lose weight was even greater in magnitude when assessed in the on-treatment analysis (<2% weight loss: semaglutide, 0.83; placebo, 1.02; ETR, 0.81; Table S8).

### Cardiovascular Outcomes by Change in hsCRP Level During SELECT

**Figure 6.**
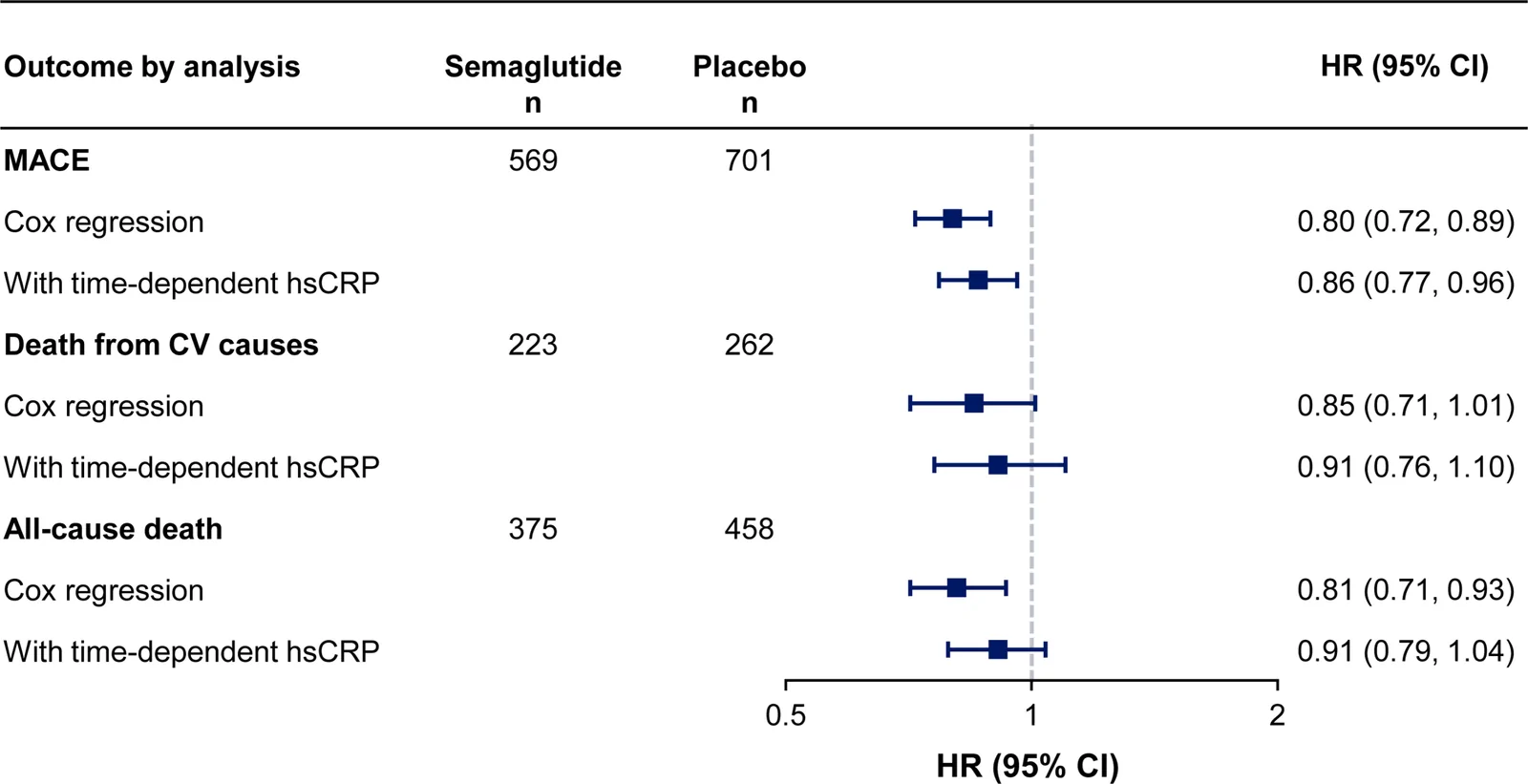
**Forest plot of time to event analyses of selected cardiovascular outcomes by time-dependent changes in high-sensitivity CRP during the trial.** Data are the estimated HRs, analyzed using a Cox proportional hazards model with treatment group as a categorical fixed factor, using in-trial data from the observation period. The analysis with time-dependent hsCRP changes also included baseline low-density lipoprotein cholesterol level, statin use, and baseline log hsCRP as covariates, and log ratio-to-baseline hsCRP as a time-dependent covariate. n denotes the number of patients who had an event within these categories. MACE is unadjusted for the group-sequential design. CV indicates cardiovascular; HR, hazard ratio; hsCRP, high-sensitivity C-reactive protein; and MACE, major adverse cardiovascular events.

The relationship between hsCRP changes during the trial and the reduction in MACE, death from cardiovascular causes, and all-cause death among treatment groups was studied by analyzing the attenuation in the semaglutide-associated reduction in cardiovascular outcomes when factoring in hsCRP changes as a time-varying covariate (Figure 6). In such analyses, a variable that fully marks or mediates a treatment’s beneficial effect on the distribution of the time to an event will drive the adjusted treatment effect estimate toward the null hypothesis of an HR of 1.0. As shown, after factoring in time-dependent changes in hsCRP levels, the decrease in MACE observed in SELECT on the Cox regression analysis of HR, 0.80 (95% CI, 0.72, 0.89) with semaglutide versus placebo was attenuated to HR, 0.86 (95% CI, 0.77, 0.96). Similar attenuation patterns were observed when comparing the overall trial effect without and with adjustment for hsCRP changes, respectively, on death from cardiovascular causes (HR, 0.85 [95% CI, 0.71, 1.01] versus HR, 0.91 [95% CI, 0.76, 1.10]) and all-cause death (HR, 0.81 [95% CI, 0.71, 0.93] versus HR, 0.91 [95% CI, 0.79, 1.04]), although several of these measures crossed the line of identity (Figure 6). Such results can be characterized as suggesting that semaglutide-mediated changes in hsCRP level mark effect contributions of 32% on MACE, 42% on death from cardiovascular causes, and 45% on all-cause mortality.

In further analysis using joint linear continuous modeling of risk of MACE by change in body weight and baseline body weight or change in hsCRP level and baseline hsCRP level, greater reduction in hsCRP level at week 104 was strongly prognostic of lower risk of MACE in a linear manner, with marginally significantly steeper slopes (*P*_interaction_=0.048) in the semaglutide (HR, 0.93 per 25% decrease in hsCRP [95% CI, 0.91, 0.95]; *P*<0.001) versus placebo arms (HR, 0.96 per 25% decrease in hsCRP level [95% CI, 0.94, 0.98]; *P*<0.001). Similar effects for each treatment arm were observed post hoc in the male subgroup; however, in the female subgroup, the association between proportionate change in hsCRP level and risk of MACE showed no clear linear trend and had weak evidence (*P=*0.045) for nonlinear associations (Table S9). MACE incidence rates by treatment arm and HRs across treatment arms are also presented within categories of percent change in body weight and proportionate change of hsCRP level (Table S10). A graphical display of smoothed MACE incidence rates by categorical changes in body weight and hsCRP level is provided by treatment arm and sex (Figure S6), which makes evident the greater imprecision afforded by the smaller sample sizes in the female subgroup (24% of all SELECT patients, 21% of MACE). Similar linear continuous modeling of risk of in-trial MACE by time-varying weight loss or time-varying decrease in hsCRP level without imputation provided similar results (Table S11).

## Discussion

In SELECT, the GLP-1 RA semaglutide significantly reduced MACE by 20% in patients with established ASCVD and overweight or obesity but not diabetes, compared with placebo.^20^ In this prespecified SELECT analysis, higher baseline hsCRP values correlated positively with multiple measures of increasing adiposity and were prognostic of an increased risk of MACE, cardiovascular death, and all-cause death in the placebo and semaglutide treatment groups. The semaglutide effect on MACE reduction was homogenous across baseline hsCRP values, but greater body weight loss was associated with larger hsCRP level reductions. Among semaglutide-treated patients, hsCRP level decreases were evident before full dose escalation, before significant weight loss, and among those with minimal to absent body weight loss (<2%). Body weight loss in patients receiving placebo was not associated with a similar magnitude of hsCRP reduction as in those receiving semaglutide. Further analysis of the relationship between MACE outcomes and measurements of body weight (baseline body weight and change in body weight) and hsCRP (baseline hsCRP level and change in hsCRP levels) suggests that neither of these measures alone fully accounts for the benefits seen in SELECT. Modeling outcomes among semaglutide versus placebo groups indicates that change in hsCRP levels may partially contribute to but not fully explain the reduction in MACE seen in SELECT. These novel findings regarding baseline hsCRP levels and semaglutide effects on hsCRP levels over time in patients with ASCVD and obesity but not diabetes provide additional, unique data regarding hsCRP level as a biomarker for cardiovascular risk and semaglutide effects on inflammation, even if within the context and limitations of this prespecified secondary analysis.

Patients in SELECT randomized to semaglutide had significant, rapid, and sustained reductions in hsCRP level (38%) over 104 weeks compared with those assigned to placebo. These semaglutide effects occurred in patients with median baseline hsCRP levels below the threshold of 2 mg/L (median: semaglutide, 1.87 mg/L; placebo, 1.80 mg/L) cited in guidelines as identifying residual risk from systemic inflammation.^24,25^ The baseline hsCRP levels in SELECT were significantly lower than in previous semaglutide studies, possibly as a result of the specific nature of the SELECT population and their management. In STEP-HFpEF (Research Study to Investigate How Well Semaglutide Works in People Living With Heart Failure and Obesity), which investigated semaglutide 2.4 mg versus placebo in 529 patients with heart failure with preserved ejection fraction, the median baseline hsCRP level was 3.8 mg/L, with semaglutide exerting an estimated treatment difference for hsCRP of −39%, similar to that observed in SELECT.^26^ In previous placebo-controlled weight loss intervention trials with semaglutide 2.4 mg, median baseline hsCRP values were 3.9 mg/L (STEP 1),^27^ 3.4 mg/L (STEP 2),^28^ and 4.5 mg/L (STEP 3),^29^ with an estimated treatment difference (95% CI) for hsCRP level with semaglutide of −44% (–49, −39), −39% (–46, −30), and −48% (–55, −39), respectively. These weight loss–focused studies demonstrated greater body weight reduction than that observed in SELECT,^27–29^ which involved no body weight loss guidance or support. Semaglutide reduced hsCRP levels significantly in SELECT, despite patients having lower baseline hsCRP levels than in the other trials, lower baseline LDL-C levels (median 78 mg/dL) than in most cholesterol-lowering trials, and extensive use of statins (88%), which themselves lower hsCRP levels.^5^ Semaglutide decreased hsCRP levels consistently across all patient subgroups and independent of multiple clinical measures, albeit with a slightly greater effect in women, in keeping with other studies in which the effect of semaglutide on hsCRP levels was investigated.^30^

This SELECT data analysis extends evidence regarding the interaction between overweight or obesity and inflammation, including how body weight loss alters hsCRP levels in a secondary prevention ASCVD cohort. Among patients in SELECT grouped according to baseline hsCRP levels (<2 mg/L, 2–<10 mg/L, or ≥10 mg/L), a stepwise increase in baseline BMI, body weight, and waist circumference is seen. Increasing weight loss in the semaglutide group was associated with greater reductions in ratio-to-baseline hsCRP, a trend absent in the placebo group, even among the small number of placebo-treated patients with greater body weight loss (≥10%–15%; n=384); among placebo-treated patients who lost ≥15% of their body weight (n=125), a modest decrease in ratio-to-baseline hsCRP occurred (ratio-to-baseline hsCRP of 0.73). Whether such significant body weight loss in this subgroup of patients receiving placebo involved other health issues or factors, such as statin titration or initiation of other medications, cannot be excluded. In contrast to semaglutide treatment, patients on placebo who lost the most body weight also experienced the greatest increase in risk of MACE.

The temporal relationship among semaglutide, hsCRP changes, and body weight loss is noteworthy. Semaglutide resulted in a rapid decrease in hsCRP levels, with approximately half of the overall decline in ratio-to-baseline hsCRP that would occur over the 104-week assessment period evident by 8 weeks; at this time point, patients assigned to semaglutide had only lost ≈3% of their body weight and had not yet undergone dose escalation to the full 2.4 mg dose.^20,31^ This pattern suggests that some semaglutide effects on hsCRP level may precede major changes in body weight or occur with minimal weight loss. Similarly, although hsCRP level declined more with greater body weight loss, decreases in the ratio-to-baseline hsCRP were also evident among semaglutide-treated patients with minimal or no weight loss, compared with placebo. The rapid effect of semaglutide on hsCRP levels before substantial body weight loss could still involve changes in adipose tissue or other biologic mechanisms that might precede or occur separately from body weight changes, with multiple inputs affecting circulating levels of hsCRP, which is produced by both adipose tissue and the liver.^32^ Other potential explanations, such as semaglutide-induced changes in dietary patterns or other adipose-independent effects, whether GLP-1 receptor–dependent or –independent, remain unclear, cannot be resolved in this clinical trial substudy, and warrant further investigation.

Inflammatory marker patterns observed in SELECT may have relevant clinical implications. Increasing baseline hsCRP levels among SELECT participants, who all had established ASCVD and overweight or obesity, reveal a progressive increased risk in MACE, cardiovascular death, and all-cause death, despite median baseline hsCRP levels <2.0 mg/L, as evident in both treatment groups. Assignment to semaglutide reduced the risk of MACE compared with placebo similarly across all baseline hsCRP groups, including those with baseline levels <2 mg/L. Such hsCRP findings may align with other cardiovascular risk factor data that demonstrate a continuous relationship with cardiovascular risk, even at lower levels, as seen with LDL-C level and its treatment.^33,34^ Particularly striking in SELECT was the increase in catastrophic clinical events of cardiovascular death and all-cause death in those with the highest baseline hsCRP levels (≥10 mg/L; Figure 3) in these patients with chronic ASCVD. This finding, consistent with recent data from several major contemporary studies,^35–39^ suggests that hsCRP measurements may help identify patients with ASCVD with overweight or obesity at a higher risk of such MACE, enabling consideration of measures that might offset such outcomes, including semaglutide.

The data provided on changes in hsCRP levels in SELECT may offer some insight into mechanisms contributing to the effects of semaglutide on decreasing MACE in SELECT, which remain unclear. Semaglutide treatment was associated with modest improvements in triglycerides, systolic blood pressure, and glucose levels, all of which are also associated with inflammation, whether as a cause or consequence. The effects of semaglutide on inflammation may involve a component that occurs independent of body weight loss, whether through direct or indirect GLP-1 receptor activation, through action on the immune system, or other mechanisms. This clinical trial substudy can neither prove nor disprove modification of inflammation as contributing to the benefits of semaglutide in SELECT, but detailed analysis of the data provided here suggests that the decrease in MACE seen with semaglutide versus placebo in SELECT was only partially attenuated after accounting for hsCRP changes. As such, a decrease in inflammation, as indicated by hsCRP changes, may have contributed to some but not all semaglutide benefits seen in SELECT. The data provided show that the strongest association between body weight loss and changes in hsCRP level occurred in the semaglutide group and were not seen in placebo-treated patients. Neither SELECT nor this SELECT substudy can disentangle whether the stronger associations seen with semaglutide on body weight loss and change in hsCRP level involve semaglutide acting on a common pathway that modulates both adiposity and inflammation, semaglutide action on separate pathways related to body weight and inflammation, or a combination of these 2 hypotheses. Further insight into inflammation in ASCVD should come from ongoing trials of agents that directly modify inflammatory pathways, such as IL-6 inhibition, in which far larger hsCRP percent reductions are observed and where inhibition of innate immune pathways is better understood.^40,41^

Several limitations exist in this prespecified analysis of inflammation in SELECT. hsCRP is a single inflammatory biomarker and established as not being a pathogenic mediator. Assessing levels of proteins directly involved in inflammatory processes in response to GLP-1 RA activation may provide greater understanding and precision regarding inflammatory contributions to obesity-associated cardiovascular risk and how body weight loss modifies that risk. Nevertheless, the reproducibility, reliability, and previous studies of hsCRP, as well as its incorporation into guidelines, support its investigation here. No specific adjustment in SELECT was made for multiplicity. As noted, SELECT was not a weight loss intervention trial, with less body weight loss observed than in other semaglutide studies, which may have limited potential hsCRP changes. As a sensitive measure, hsCRP levels can be altered by multiple factors, which might generate misleading findings; the consistent hsCRP results seen using multiple approaches, including assessment of hsCRP tertiles, various clinical measures, and different cardiovascular outcomes, as well as hsCRP measurements at baseline and over time, all undertaken in this large cohort and compared with placebo, offset such concerns.

Taken together, this prespecified analysis of patients enrolled in SELECT reveals an association between higher baseline hsCRP levels and increased risk of subsequent MACE in a cohort with established ASCVD and overweight or obesity but no diabetes. Notably, this cohort had relatively low baseline hsCRP levels, extensive statin use, and reasonably controlled LDL-C levels. Baseline hsCRP levels were higher across categories of increasing baseline BMI, whereas greater body weight loss with semaglutide was associated with larger decreases in hsCRP. These effects were evident early after treatment initiation, before major weight loss occurred, and were also observed among semaglutide-treated patients who did not lose body weight, all compared with placebo. The association between higher baseline hsCRP level and subsequent cardiovascular events in SELECT patients supports a complex relationship among inflammation, obesity, and ASCVD, as well as the potential utility of inflammatory biomarkers, such as hsCRP level, in helping identify residual cardiovascular risk in patients with obesity and ASCVD. Furthermore, semaglutide use and its accompanying body weight loss were associated with decreases in hsCRP levels and MACE, suggesting a reduction in inflammation as a potential contributor to the benefits of semaglutide seen in SELECT.

## Article Information

### Acknowledgments

Editorial support was provided by Eleanor Finn, PhD, of Apollo, OPEN Health Communications, and funded by Novo Nordisk A/S, in accordance with Good Publication Practice guidelines (www.ismpp.org/gpp-2022).

### Sources of Funding

The trial was funded by Novo Nordisk A/S.

### Disclosures

Dr Plutzky declares having received consulting honoraria from Altimmune, Amgen, Boehringer Ingelheim, Corcept, Esperion Therapeutics, Merck, MJH Life Sciences, New Amsterdam, Novartis, and Novo Nordisk; has received grants, paid to his institution, from Boehringer Ingelheim and Novartis; and holds the position of Director, Preventive Cardiology, at Brigham and Women’s Hospital. Dr Bogdański declares serving on advisory panels for Bausch Health, Eli Lilly, and Novo Nordisk; and has received speaker honoraria from Boehringer Ingelheim, Lilly, Mylan, Novo Nordisk, and Sanofi. Prof Colhoun declares serving on advisory panels for Bayer and Novo Nordisk; receiving research funding from IQVIA, Roche Pharmaceuticals, and Sanofi; receiving grants from the Chief Scientist Office, Diabetes UK, European Commission, Juvenile Diabetes Research Foundation, and Medical Research Council; serving on a speaker’s bureau for Novo Nordisk; and holding stock in Bayer and Roche Pharmaceuticals. Dr Dagdelen declares participating in advisory board panels for Bayer, Novo Nordisk, and Sanofi. Prof Deanfield declares having received consulting honoraria from Aegerion, Amgen, Bayer, Boehringer Ingelheim, Merck, Novartis, Novo Nordisk, Pfizer, Sanofi, and Takeda; and research grants from Aegerion, the British Heart Foundation, Colgate, Medical Research Council (UK), MSD, National Institute for Health and Care Research, Pfizer, Public Health England, and Roche. Prof Emerson declares having served as a consultant for Novo Nordisk through participation on steering committees for cardiovascular outcome trials of semaglutide in obesity and overweight (SELECT) and in type 2 diabetes (SOUL [Semaglutide Cardiovascular Outcomes Trial]), with an earlier role on a cardiovascular outcomes trial of insulin degludec. Dr Hovingh declares being an employee of and shareholder in Novo Nordisk. Dr Kahn declares having received consulting honoraria from Amgen, Anji Pharmaceuticals, Boehringer Ingelheim, Biomea Fusion, Eli Lilly, Merck, Novo Nordisk, and Oramed; stock options from Altpep; and research support from Corcept Therapeutics. Dr Ekström declares being a full-time employee of and shareholder in Novo Nordisk. Dr Latkovskis has given talks, attended conferences, received consultancy fees, or participated in trials sponsored by 89bio, Abbott Laboratories, Amgen, AstraZeneca, Berlin Chemie, Bayer, Boehringer Ingelheim, GlaxoSmithKline, Grindex, KRKA, MSD, Mylan, Novartis, Novo Nordisk, Pfizer, Roche Diagnostics, Sanofi, Servier Laboratories, Siemens Laboratories, Swixx Biopharma, and Zentiva. Dr Lehrke declares scientific support from Boehringer Ingelheim, MSD, and Novo Nordisk; speaker honoraria from Abiomed, Amgen, AstraZeneca, BMS, Boehringer Ingelheim, Daiichi Sankyo, Lilly, MSD, Novo Nordisk, and Sanofi; and advisory support from Amgen, AstraZeneca, Boehringer Ingelheim, Lilly, MSD, Novo Nordisk, and Sanofi. Dr Hardt-Lindberg declares being a full-time employee of and shareholder in Novo Nordisk. Prof Lingvay declares having received research grants from Boehringer Ingelheim, Merck, Mylan Pharmaceuticals Inc., Novo Nordisk, Pfizer, and Sanofi US Services; has served as a consultant for AstraZeneca, Bayer Healthcare Pharmaceuticals Inc., Biomea, Boehringer Ingelheim, Carmot, Eli Lilly and Company, Intarcia, Intercept Pharmaceuticals, Inc., Janssen Global Services, Johnson & Johnson Medical Devices & Diagnostics Group–Latin America, MannKind Corporation, Merck, Novo Nordisk, Pfizer, Sanofi US Services, Shionogi, Structure Therapeutics, Target Pharma, Valeritas, and Zealand Pharma; and has received travel expenses from Boehringer Ingelheim, Eli Lilly and Company, Johnson & Johnson Medical Devices & Diagnostics Group–Latin America, Novo Nordisk, Sanofi US Services, and Zealand Pharma. Dr Nicholls has received research support from AstraZeneca, Amgen, Anthera, CSL Behring, Cerenis, Cyclarity, Eli Lilly, Esperion, Resverlogix, New Amsterdam Pharma, Novartis, Infraredx, and Sanofi-Regeneron, and is a consultant for Amgen, Akcea, AstraZeneca, Boehringer Ingelheim, CSL Behring, Daiichi Sankyo, Eli Lilly, Esperion, Kowa, Merck, Takeda, Pfizer, Sanofi-Regeneron, Vaxxinity, CSL Seqiris, and Novo Nordisk. Dr Kalayci Oral declares being a full-time employee of and shareholder in Novo Nordisk. Dr Terns has received fees from Amgen, AstraZeneca, Boehringer Ingelheim, and Novo Nordisk. Dr Rasmussen declares being a full-time employee of and shareholder in Novo Nordisk. Dr Ridker has received institutional research grant support from the National Heart, Lung, and Blood Institute, Novartis, and Novo Nordisk (to evaluate the role of anti-inflammatory agents, including methotrexate, interleukin-1 inhibitors, and interleukin-6 inhibitors), as well as Amarin, Esperion, Kowa, and Pfizer; has served as a consultant to Agepha, Ardelyx, Cardio Therapeutics, CSL Behring, Flame, Horizon Therapeutics, Janssen, Novartis, Novo Nordisk, and Zomagen (entities developing anti-inflammatory therapies, including colchicine, interleukin-1 inhibitors, interleukin-6 inhibitors, and agents that potentially target or interact with the NLRP3 inflammasome); has served as a consultant to AstraZeneca, Boehringer Ingelheim, Civi Biopharma, Cytokinetics, Eli Lilly, GlaxoSmithKline, Montai Health, New Amsterdam, RTI, SOCAR, and Health Outlook; has minority shareholder equity positions in Angiowave, Bitteroot Bio, and Upperton; and receives compensation for service on the Baim Institute, Leducq Foundation, Paris FR, and the Peter Munk Advisory Board (University of Toronto). Prof Ryan declares having received consulting honoraria from Altimmune, Amgen, AstraZeneca, Biohaven, Boehringer Ingelheim, Calibrate, Carmot Therapeutics, CinRx, Eli Lilly, eMed, Epitomee, Gila Therapeutics, Ifa Celtic, Novo Nordisk, Pfizer, Regeneron, Rhythm, Roche Pharmaceuticals, Scientific Intake, Wondr Health, and Zealand; and received stock options from Calibrate, Epitomee, Scientific Intake, and Xeno Bioscience. Prof Lincoff declares having received research grants paid to his institution from AbbVie, AstraZeneca, CSL Behring, Eli Lilly and Company, Esperion Therapeutics, and Novartis; and has received consultancy fees from Akebia Therapeutics, Alnylam Pharmaceuticals, Ardelyx, Canary Cure, Eli Lilly and Company, Entity, FibroGen, GlaxoSmithKline, Intarcia, Medtronic Vascular, Novartis Pharmaceuticals Corporation, Novo Nordisk, Provention Bio, and ReCor Medical.

### Supplemental Material

CONSORT Guidelines

Methods

Tables S1–S11

Figures S1–S6

## Supplementary Material

## References

[1] Hansson GK. Inflammation, atherosclerosis, and coronary artery disease. N Engl J Med. 2005;352:1685–1695. doi: 10.1056/NEJMra043430 15843671 10.1056/NEJMra043430

[2] Libby P Buring JE Badimon L Hansson GK Deanfield J Bittencourt MS Tokgozoglu L Lewis EF. Atherosclerosis. Nat Rev Dis Primers. 2019;5:56. doi: 10.1038/s41572-019-0106-z 31420554 10.1038/s41572-019-0106-z

[3] Visseren FLJ Mach F Smulders YM Carballo D Koskinas KC Back M Benetos A Biffi A Boavida JM Capodanno D; ESC National Cardiac Societies. 2021 ESC guidelines on cardiovascular disease prevention in clinical practice. Eur Heart J. 2021;42:3227–3337. doi: 10.1093/eurheartj/ehab484 34458905 10.1093/eurheartj/ehab484

[4] Virani SS Newby LK Arnold SV Bittner V Brewer LC Demeter SH Dixon DL Fearon WF Hess B Johnson HM; Writing Committee Members. 2023 AHA/ACC/ACCP/ASPC/NLA/PCNA guideline for the management of patients with chronic coronary disease: a report of the American Heart Association/American College of Cardiology joint committee on clinical practice guidelines. J Am Coll Cardiol. 2023;82:833–955. doi: 10.1016/j.jacc.2023.04.003 37480922 10.1016/j.jacc.2023.04.003

[5] Ridker PM Danielson E Fonseca FA Genest J Gotto AM Jr Kastelein JJ Koenig W Libby P Lorenzatti AJ MacFadyen JG; JUPITER Study Group. Rosuvastatin to prevent vascular events in men and women with elevated C-reactive protein. N Engl J Med. 2008;359:2195–2207. doi: 10.1056/NEJMoa0807646 18997196 10.1056/NEJMoa0807646

[6] Ridker PM Everett BM Thuren T MacFadyen JG Chang WH Ballantyne C Fonseca F Nicolau J Koenig W Anker SD; CANTOS Trial Group. Antiinflammatory therapy with canakinumab for atherosclerotic disease. N Engl J Med. 2017;377:1119–1131. doi: 10.1056/NEJMoa1707914 28845751 10.1056/NEJMoa1707914

[7] Tardif JC Kouz S Waters DD Bertrand OF Diaz R Maggioni AP Pinto FJ Ibrahim R Gamra H Kiwan GS. Efficacy and safety of low-dose colchicine after myocardial infarction. N Engl J Med. 2019;381:2497–2505. doi: 10.1056/NEJMoa1912388 31733140 10.1056/NEJMoa1912388

[8] Jolly SS d’Entremont MA Lee SF Mian R Tyrwhitt J Kedev S Montalescot G Cornel JH Stanković G Moreno R; CLEAR Investigators. Colchicine in acute myocardial infarction. N Engl J Med. 2025;392:633–642. doi: 10.1056/NEJMoa2405922 39555823 10.1056/NEJMoa2405922

[9] Klein S Burke LE Bray GA Blair S Allison DB Pi-Sunyer X Hong Y Eckel RH; American Heart Association Council on Nutrition, Physical Activity, and Metabolism. Clinical implications of obesity with specific focus on cardiovascular disease: a statement for professionals from the American Heart Association Council on Nutrition, Physical Activity, and Metabolism: endorsed by the American College of Cardiology Foundation. Circulation. 2004;110:2952–2967. doi: 10.1161/01.CIR.0000145546.97738.1E 15509809 10.1161/01.CIR.0000145546.97738.1E

[10] Yudkin JS Stehouwer CD Emeis JJ Coppack SW. C-reactive protein in healthy subjects: associations with obesity, insulin resistance, and endothelial dysfunction: a potential role for cytokines originating from adipose tissue? Arterioscler Thromb Vasc Biol. 1999;19:972–978. doi: 10.1161/01.atv.19.4.972 10195925 10.1161/01.atv.19.4.972

[11] Hak AE Stehouwer CD Bots ML Polderman KH Schalkwijk CG Westendorp IC Hofman A Witteman JC. Associations of C-reactive protein with measures of obesity, insulin resistance, and subclinical atherosclerosis in healthy, middle-aged women. Arterioscler Thromb Vasc Biol. 1999;19:1986–1991. doi: 10.1161/01.atv.19.8.1986 10446082 10.1161/01.atv.19.8.1986

[12] Visser M Bouter LM McQuillan GM Wener MH Harris TB. Elevated C-reactive protein levels in overweight and obese adults. JAMA. 1999;282:2131–2135. doi: 10.1001/jama.282.22.2131 10591334 10.1001/jama.282.22.2131

[13] Kawai T Autieri MV Scalia R. Adipose tissue inflammation and metabolic dysfunction in obesity. Am J Physiol Cell Physiol. 2021;320:C375–C391. doi: 10.1152/ajpcell.00379.2020 33356944 10.1152/ajpcell.00379.2020 PMC8294624

[14] Belza A Toubro S Stender S Astrup A. Effect of diet-induced energy deficit and body fat reduction on high-sensitive CRP and other inflammatory markers in obese subjects. Int J Obes (Lond). 2009;33:456–464. doi: 10.1038/ijo.2009.27 19238154 10.1038/ijo.2009.27

[15] Nicklas JM Sacks FM Smith SR LeBoff MS Rood JC Bray GA Ridker PM. Effect of dietary composition of weight loss diets on high-sensitivity c-reactive protein: the Randomized POUNDS LOST trial. Obesity (Silver Spring). 2013;21:681–689. doi: 10.1002/oby.20072 23712970 10.1002/oby.20072 PMC3671388

[16] Selvin E Paynter NP Erlinger TP. The effect of weight loss on C-reactive protein: a systematic review. Arch Intern Med. 2007;167:31–39. doi: 10.1001/archinte.167.1.31 17210875 10.1001/archinte.167.1.31

[17] Marso SP Daniels GH Brown-Frandsen K Kristensen P Mann JF Nauck MA Nissen SE Pocock S Poulter NR Ravn LS; LEADER Steering Committee. Liraglutide and cardiovascular outcomes in type 2 diabetes. N Engl J Med. 2016;375:311–322. doi: 10.1056/NEJMoa1603827 27295427 10.1056/NEJMoa1603827 PMC4985288

[18] Marso SP Bain SC Consoli A Eliaschewitz FG Jódar E Leiter LA Lingvay I Rosenstock J Seufert J Warren ML; SUSTAIN-6 Investigators. Semaglutide and cardiovascular outcomes in patients with type 2 diabetes. N Engl J Med. 2016;375:1834–1844. doi: 10.1056/NEJMoa1607141 27633186 10.1056/NEJMoa1607141

[19] Verma S Bhatta M Davies M Deanfield JE Garvey WT Jensen C Kandler K Kushner RF Rubino DM Kosiborod MN. Effects of once-weekly semaglutide 2.4 mg on C-reactive protein in adults with overweight or obesity (STEP 1, 2, and 3): exploratory analyses of three randomised, double-blind, placebo-controlled, phase 3 trials. EClinicalMedicine. 2023;55:101737. doi: 10.1016/j.eclinm.2022.101737 36467859 10.1016/j.eclinm.2022.101737 PMC9713290

[20] Lincoff AM Brown-Frandsen K Colhoun HM Deanfield J Emerson SS Esbjerg S Hardt-Lindberg S Hovingh GK Kahn SE Kushner RF; SELECT Trial Investigators. Semaglutide and cardiovascular outcomes in obesity without diabetes. N Engl J Med. 2023;389:2221–2232. doi: 10.1056/NEJMoa2307563 37952131 10.1056/NEJMoa2307563

[21] Ryan DH Lingvay I Colhoun HM Deanfield J Emerson SS Kahn SE Kushner RF Marso S Plutzky J Brown-Frandsen K. Semaglutide effects on cardiovascular outcomes in people with overweight or obesity (SELECT) rationale and design. Am Heart J. 2020;229:61–69. doi: 10.1016/j.ahj.2020.07.008 32916609 10.1016/j.ahj.2020.07.008

[22] Lingvay I Brown-Frandsen K Colhoun HM Deanfield J Emerson SS Esbjerg S Hardt-Lindberg S Hovingh GK Kahn SE Kushner RF; SELECT Study Group. Semaglutide for cardiovascular event reduction in people with overweight or obesity: SELECT study baseline characteristics. Obesity (Silver Spring). 2023;31:111–122. doi: 10.1002/oby.23621 36502289 10.1002/oby.23621 PMC10107832

[23] Borgan Ø. Aalen–Johansen estimator. In: Wiley StatsRef: Statistics Reference Online. 2018; 1–13. doi: 10.1002/9781118445112.stat06001.pub2

[24] Grundy SM Stone NJ Bailey AL Beam C Birtcher KK Blumenthal RS Braun LT de Ferranti S Faiella-Tommasino J Forman DE. 2018 AHA/ACC/AACVPR/AAPA/ABC/ACPM/ADA/AGS/APhA/ASPC/NLA/PCNA guideline on the management of blood cholesterol: a report of the American College of Cardiology/American Heart Association task force on clinical practice guidelines. Circulation. 2019;139:e1082–e1143. doi: 10.1161/CIR.0000000000000625 30586774 10.1161/CIR.0000000000000625 PMC7403606

[25] Pearson GJ Thanassoulis G Anderson TJ Barry AR Couture P Dayan N Francis GA Genest J Gregoire J Grover SA. 2021 Canadian Cardiovascular Society guidelines for the management of dyslipidemia for the prevention of cardiovascular disease in adults. Can J Cardiol. 2021;37:1129–1150. doi: 10.1016/j.cjca.2021.03.016 33781847 10.1016/j.cjca.2021.03.016

[26] Kosiborod MN Abildstrøm SZ Borlaug BA Butler J Rasmussen S Davies M Hovingh GK Kitzman DW Lindegaard ML Møller DV; STEP-HFpEF Trial Committees and Investigators. Semaglutide in patients with heart failure with preserved ejection fraction and obesity. N Engl J Med. 2023;389:1069–1084. doi: 10.1056/NEJMoa2306963 37622681 10.1056/NEJMoa2306963

[27] Wilding JPH Batterham RL Calanna S Davies M Van Gaal LF Lingvay I McGowan BM Rosenstock J Tran MTD Wadden TA; STEP 1 Study Group. Once-weekly semaglutide in adults with overweight or obesity. N Engl J Med. 2021;384:989–1002. doi: 10.1056/NEJMoa2032183 33567185 10.1056/NEJMoa2032183

[28] Davies M Faerch L Jeppesen OK Pakseresht A Pedersen SD Perreault L Rosenstock J Shimomura I Viljoen A Wadden TA; STEP 2 Study Group. Semaglutide 2.4 mg once a week in adults with overweight or obesity, and type 2 diabetes (STEP 2): a randomised, double-blind, double-dummy, placebo-controlled, phase 3 trial. Lancet. 2021;397:971–984. doi: 10.1016/S0140-6736(21)00213-0 33667417 10.1016/S0140-6736(21)00213-0

[29] Wadden TA Bailey TS Billings LK Davies M Frias JP Koroleva A Lingvay I O’Neil PM Rubino DM Skovgaard D; STEP 3 Investigators. Effect of subcutaneous semaglutide vs placebo as an adjunct to intensive behavioral therapy on body weight in adults with overweight or obesity: the STEP 3 randomized clinical trial. JAMA. 2021;325:1403–1413. doi: 10.1001/jama.2021.1831 33625476 10.1001/jama.2021.1831 PMC7905697

[30] Verma S Butler J Borlaug BA Davies M Kitzman DW Shah SJ Petrie MC Barros E Rönnbäck C Vestergaard LS; STEP-HFpEF Trial Committees and Investigators. Efficacy of semaglutide by sex in obesity-related heart failure with preserved ejection fraction: STEP-HFpEF trials. J Am Coll Cardiol. 2024;84:773–785. doi: 10.1016/j.jacc.2024.06.001 38913003 10.1016/j.jacc.2024.06.001

[31] Ryan DH Lingvay I Deanfield J Kahn SE Barros E Burguera B Colhoun HM Cercato C Dicker D Horn DB. Long-term weight loss effects of semaglutide in obesity without diabetes in the SELECT trial. Nat Med. 2024;30:2049–2057. doi: 10.1038/s41591-024-02996-7 38740993 10.1038/s41591-024-02996-7 PMC11271387

[32] Neeland IJ Ross R Despres JP Matsuzawa Y Yamashita S Shai I Seidell J Magni P Santos RD Arsenault B; International Atherosclerosis Society. Visceral and ectopic fat, atherosclerosis, and cardiometabolic disease: a position statement. Lancet Diabetes Endocrinol. 2019;7:715–725. doi: 10.1016/S2213-8587(19)30084-1 31301983 10.1016/S2213-8587(19)30084-1

[33] Baigent C Blackwell L Emberson J Holland LE Reith C Bhala N Peto R Barnes EH Keech A Simes J; Cholesterol Treatment Trialists’ (CTT) Collaboration. Efficacy and safety of more intensive lowering of LDL cholesterol: a meta-analysis of data from 170,000 participants in 26 randomised trials. Lancet. 2010;376:1670–1681. doi: 10.1016/S0140-6736(10)61350-5 21067804 10.1016/S0140-6736(10)61350-5 PMC2988224

[34] Silverman MG Ference BA Im K Wiviott SD Giugliano RP Grundy SM Braunwald E Sabatine MS. Association between lowering LDL-C and cardiovascular risk reduction among different therapeutic interventions: a systematic review and meta-analysis. JAMA. 2016;316:1289–1297. doi: 10.1001/jama.2016.13985 27673306 10.1001/jama.2016.13985

[35] Kaura A Hartley A Panoulas V Glampson B Shah ASV Davies J Mulla A Woods K Omigie J Shah AD. Mortality risk prediction of high-sensitivity C-reactive protein in suspected acute coronary syndrome: a cohort study. PLoS Med. 2022;19:e1003911. doi: 10.1371/journal.pmed.1003911 35192610 10.1371/journal.pmed.1003911 PMC8863282

[36] Zhang L He G Huo X Tian A Ji R Pu B Peng Y. Long-term cumulative high-sensitivity c-reactive protein and mortality among patients with acute heart failure. J Am Heart Assoc. 2023;12:e029386. doi: 10.1161/JAHA.123.029386 37776214 10.1161/JAHA.123.029386 PMC10727254

[37] Gedebjerg A Bjerre M Kjaergaard AD Nielsen JS Rungby J Brandslund I Maeng M Beck-Nielsen H Vaag A Sørensen HT. CRP, C-peptide, and risk of first-time cardiovascular events and mortality in early type 2 diabetes: a Danish cohort study. Diabetes Care. 2023;46:1037–1045. doi: 10.2337/dc22-1353 36930691 10.2337/dc22-1353

[38] Ridker PM Bhatt DL Pradhan AD Glynn RJ MacFadyen JG Nissen SE; PROMINENT, REDUCE-IT, and STRENGTH Investigators. Inflammation and cholesterol as predictors of cardiovascular events among patients receiving statin therapy: a collaborative analysis of three randomised trials. Lancet. 2023;401:1293–1301. doi: 10.1016/S0140-6736(23)00215-5 36893777 10.1016/S0140-6736(23)00215-5

[39] Ridker PM Lei L Louie MJ Haddad T Nicholls SJ Lincoff AM Libby P Nissen SE; CLEAR Outcomes Investigators. Inflammation and cholesterol as predictors of cardiovascular events among 13 970 contemporary high-risk patients with statin intolerance. Circulation. 2024;149:28–35. doi: 10.1161/CIRCULATIONAHA.123.066213 37929602 10.1161/CIRCULATIONAHA.123.066213 PMC10752259

[40] Ridker PM Devalaraja M Baeres FMM Engelmann MDM Hovingh GK Ivkovic M Lo L Kling D Pergola P Raj D; RESCUE Investigators. IL-6 inhibition with ziltivekimab in patients at high atherosclerotic risk (RESCUE): a double-blind, randomised, placebo-controlled, phase 2 trial. Lancet. 2021;397:2060–2069. doi: 10.1016/S0140-6736(21)00520-1 34015342 10.1016/S0140-6736(21)00520-1

[41] Zoccali C Mallamaci F. Innate immunity system in patients with cardiovascular and kidney disease. Circ Res. 2023;132:915–932. doi: 10.1161/CIRCRESAHA.122.321749 37053283 10.1161/CIRCRESAHA.122.321749

