# Supplementary material for: Effect of Semaglutide on the Inflammatory Biomarker High-Sensitivity CRP in Patients With Established Cardiovascular Disease and Overweight or Obesity in SELECT: A Prespecified Secondary Analysis
Source: Circulation. 2026 Aug 18;154(11):976–91. doi: 10.1161/CIRCULATIONAHA.125.074482 (PMC13574348; doi:10.1161/CIRCULATIONAHA.125.074482)
Supplement: Supplementary file 1 [file cir-154-976-s001.pdf]

SUPPLEMENTAL MATERIAL: Methods and Tables

CIRCULATIONAHA/2025/074482, Plutzky, et al.

"Effect of Semaglutide on the Inflammatory Biomarker High-Sensitivity CRP in Patients With Established Cardiovascular Disease and Overweight or Obesity in SELECT: A Prespecified Secondary Analysis"

Index

Supplemental Methods Overview: Complementary analysis models and statistical methods

Supplemental Tables:

Table S1. Baseline characteristics and demographics by baseline hsCRP tertiles

Table S2. Percent change in body weight and proportionate change in hsCRP by visit week on trial

Table S3. Ratio-to-baseline hsCRP at week 104 by additional baseline subgroups

Table S4. Changes from baseline to week 104 as associated with baseline measurements of body weight and hsCRP in all patients, and in male and female patients, in linear continuous models

Table S5. Joint distribution of incidence of MACE and baseline body weight or hsCRP with linear continuous modeling

Table S6. Correlations among observed changes in body weight and log hsCRP measurements while on trial at weeks 104 and 208

Table S7. Joint distribution of percent change in body weight from baseline to week 104 and the ratio of week 104 hsCRP to baseline hsCRP

Table S8. On-treatment ratio-to-baseline hsCRP by change in body weight of <2% versus ≥2% at week 104 (first on-treatment period)

Table S9. Joint linear continuous modeling of risk of MACE by week 104 change in body weight and baseline body weight or by week 104 change in hsCRP and baseline hsCRP

Table S10. Joint distribution of on-trial MACE and change in body weight or hsCRP at week 104: Incidence rates by treatment arm within categories

Table S11. Linear continuous modeling of in-trial MACE risk by time-varying body weight loss or changes in hsCRP

SUPPLEMENTAL METHODS OVERVIEW

[Additional methods provided in manuscript]

Complementary analysis models and statistical methods

In the design of SELECT, semaglutide was hypothesized to affect multiple interrelated risk factors for cardiovascular events, including body weight, glycemia, postprandial lipids, blood pressure, thrombosis, and inflammation. SELECT showed statistically significant, clinically important decreases in risk of in-trial major adverse cardiovascular events (MACE) in the semaglutide arm compared to placebo. Although not controlled for multiplicity, prespecified secondary and supportive analyses showed nominally significant improvements in biomarkers for each of the cardiovascular risk factors listed above. However, the exact mechanisms through which semaglutide decreased risk of MACE have not been, nor can they be, completely defined through secondary analyses. Nevertheless, further analysis of SELECT findings can provide insights into the effects seen and ***potential*** contributing effects, thus helping inform hypotheses about the relative contribution of various mechanistic pathways modified by semaglutide, which is of possible utility when comparing results to other trials and in guiding future studies. To this end, the supplemental results presented here describe aspects of the joint distribution of **high-sensitivity C-reactive protein (hsCRP)** as a marker of systemic inflammation, **body weight** as a marker of adiposity and a measure used clinically, and risk of MACE, as observed in SELECT. As shown here, multiple approaches were used to analyze these interactions, with complete data provided, to facilitate further consideration of the data by others and rigorous analysis of the changes seen.

hsCRP measurements over the course of SELECT were obtained at randomization, 20 weeks, 52 weeks, and then annually thereafter; on-trial changes were compared across treatment arms using the geometric mean of the proportionate change from baseline. In a subset of 5445 patients, additional hsCRP measurements were made at 4, 8, 12, and 16 weeks per protocol. Statistical analyses of the proportionate change in hsCRP across treatment arms were conducted in regression models (either linear regression or proportional hazards regression) using log-transformed hsCRP measurements. **Body weight** measurements were characterized by the cumulative percent change in body weight from baseline up to each visit week; comparisons were made across treatment arms as difference in mean percent body weight loss, as measured at randomization, every 4 weeks until week 20, and then at week 26 and every 13 weeks until the end of treatment.

Associations between body weight and hsCRP were primarily assessed using linear regression. Analyses were made of the associations between the time to a cardiovascular event included within the MACE primary endpoint, and body weight and hsCRP measurements during the study using proportional hazards regression. Additional descriptive analyses considered effects estimated within categories defined by either body weight or hsCRP, with estimation of differences in means (for weight), ratios of geometric means (for hsCRP), and inference on incidence rates per hundred years of observation time, which were analyzed using methods based on the exponential distribution. Associations between variables were primarily assessed using regression models to assess first-order (best linear) trends employing covariates modeled as linear continuous random variables. Departures from straight-line relationships were assessed inferentially by including quadratic terms in the model. As noted, some descriptive analyses of effects on risk of MACE used graphical display of moving averages of incidence rates as computed over overlapping intervals of body weight or hsCRP measurements.

While no difference in semaglutide's effect on MACE in men versus women was seen in SELECT, initial analyses of the treatment effect of semaglutide on hsCRP found nominally statistically effect modification (interaction) by sex, as seen in other glucagon-like peptide-1 receptor agonist studies. As such, results are presented for the full analysis set as well as for subgroups according to sex.

At week 104, 16% of individual patients were missing measurements for body weight or hsCRP owing to prior death (2%), prior withdrawal from the study (1%), prior loss to follow-up (1%), or failure to obtain a measure at an attended visit or sporadic missed clinic visits (12%), with the latter category potentially influenced by SELECT being conducted during the COVID-19 pandemic. The prespecified SELECT statistical analysis plan called for statistical inference of treatment effects using multiple imputation of missing data in a missing-at-random model using baseline values within treatment arms. If data were unavailable due to a missed visit, but the patient was not known to have died, the patient's missing values for each visit week were multiply imputed 500 times under a missing-at-random model that used data from each treatment arm separately, with Rubin's rules used to provide standard errors.

As described and utilized in other SELECT substudy reports (Deanfield J, Lincoff A, Kahn S et al. Semaglutide and cardiovascular outcomes by baseline and changes in adiposity measurements: a prespecified analysis of the SELECT trial. *The Lancet*, 2025; 406, 2257-2268), sensitivity analyses conducted at completion of SELECT found that the prespecified imputation scheme tended to overestimate the body weight loss in the semaglutide arm relative to available data at adjacent visits. For the analyses of hsCRP and body weight presented here, we used a broader missing-at-random model that included all available data from baseline and each postrandomization visit to impute the missing data for each patient at the particular week when this occurred. To account for patients known to have died prior to a visit week, the most recent measurement (either observed or imputed) prior to their known time of death was used. Of note, in this imputation scheme, no effort was made to impute times of death for patients who had previously withdrawn or who were lost to follow-up. Using last observation carried forward (LOCF) in this way for the on-trial measurements includes changes in hsCRP or body weight up to the earlier occurrence of the time of death or week 104. Using LOCF in this manner avoids the hypothetical assumption that patients who died would have future measurements similar to those patients still on study. The potential drawback of this approach is recognized, namely if all patients were to lose weight at a steady rate, a treatment arm with higher death rates would tend to have less body weight loss at that time point versus if they had continued in the study. Inspection of the rates of body weight loss and any differential mortality rates can help place bounds on the amount of bias potentially introduced by using LOCF in these analyses, as we have done here.

SUPPLEMENTAL Tables (S1–11)

Table S1. Baseline characteristics and demographics by baseline hsCRP tertiles

	**Baseline hsCRP tertile 1 <1.13 mg/L**		**Baseline hsCRP tertile 2 1.13 mg/L–<3.07 mg/L**		**Baseline hsCRP tertile 3 ≥3.07 mg/L**	
	**Semaglutide**	**Placebo**	**Semaglutide**	**Placebo**	**Semaglutide**	**Placebo**
**Age, years**	62.1±8.9	61.8±8.9	61.7±8.9	61.8±8.8	60.9±9.0	61.3±8.8
**Male sex, n (%)**	2316 (80.9)	2405 (82.2)	2121 (72.9)	2139 (72.6)	1868 (63.1)	1796 (62.3)
**Race or ethnic group, n (%)***						
White	2364 (82.6)	2466 (84.3)	2503 (86.0)	2513 (85.3)	2460 (83.1)	2384 (82.7)
Asian	348 (12.2)	320 (10.9)	213 (7.3)	230 (7.8)	154 (5.2)	175 (6.1)
Black	51 (1.8)	47 (1.6)	91 (3.1)	83 (2.8)	201 (6.8)	192 (6.7)
Other	67 (2.3)	68 (2.3)	73 (2.5)	95 (3.2)	112 (3.8)	107 (3.7)
Hispanic or Latino	257 (9.0)	263 (9.0)	289 (9.9)	324 (11.0)	361 (12.2)	312 (10.8)
**Body weight, kg**	93.2±14.7	94.1±15.5	96.7±17.4	96.7±17.2	99.7±19.5	99.8±20.0
**BMI, kg/m^2**	31.7±3.8	32.0±3.9	33.2±4.7	33.2±4.6	34.9±5.8	35.0±6.0
**Waist circumference, cm**	108.3±11.4	108.5±11.7	111.3±12.8	111.3±12.9	114.3±14.2	114.3±13.9
**HbA$_{1c}$, %**	5.8±0.3	5.8±0.3	5.8±0.4	5.8±0.3	5.8±0.3	5.8±0.3
**Median hsCRP level (IQR), mg/L**	0.63 (0.42–0.88)	0.64 (0.42–0.86)	1.83 (1.46–2.34)	1.81 (1.44–2.34)	5.81 (4.12–9.80)	5.81 (4.10–9.35)
**CV inclusion criteria, n (%)**						
MI only	2164 (77.0)	2178 (75.6)	1993 (69.8)	2009 (69.5)	1760 (60.8)	1725 (61.5)
Stroke only	393 (14.0)	412 (14.3)	521 (18.2)	520 (18.0)	650 (22.4)	617 (22.0)
PAD only	67 (2.4)	84 (2.9)	101 (3.5)	117 (4.0)	204 (7.0)	197 (7.0)
Two or more inclusion criteria	187 (6.7)	206 (7.2)	241 (8.4)	243 (8.4)	283 (9.8)	268 (9.5)
**eGFR, mL/min/1.73 m^2**	82.7±16.2	83.5±16.0	82.7±17.2	82.2±17.1	81.9±18.9	81.7±18.8
**Median lipid level (IQR), mmol/L**						
Total cholesterol	3.75 (3.24–4.39)	3.75 (3.25–4.38)	4.00 (3.43–4.73)	4.01 (3.46–4.76)	4.18 (3.53–5.09)	4.19 (3.53–5.10)
HDL-C	1.14 (0.98–1.36)	1.14 (0.98–1.36)	1.14 (0.97–1.36)	1.14 (0.97–1.34)	1.11 (0.94–1.31)	1.12 (0.95–1.32)
LDL-C	1.84 (1.46–2.35)	1.83 (1.45–2.32)	2.03 (1.58–2.62)	2.04 (1.61–2.63)	2.22 (1.70–2.97)	2.24 (1.71–2.98)

Triglycerides	1.42 (1.04–1.97)	1.43 (1.07–1.99)	1.53 (1.12–2.12)	1.56 (1.14–2.20)	1.59 (1.16–2.23)	1.57 (1.16–2.22)
**Systolic blood pressure, mmHg**	130.0±14.8	130.5±15.2	131.4±15.7	131.2±15.3	131.6±16.0	131.0±15.2
**Diastolic blood pressure, mmHg**	79.0±9.8	79.0±9.7	79.5±10.0	79.3±10.0	79.7±10.1	79.2±10.0
**Pulse, beats/min**	66.9±10.3	66.5±10.2	68.8±10.3	68.5±10.6	71.0±10.9	70.8±10.9

Data are mean±SD unless otherwise stated.
*See Table in manuscript for details about missing ethnicity data.
BMI, body mass index; CV, cardiovascular; eGFR, estimated glomerular filtration rate; HbA_{1c}, glycated hemoglobin; HDL-C, high-density lipoprotein cholesterol; hsCRP, high-sensitivity C-reactive protein; LDL-C, low-density lipoprotein cholesterol; MI, myocardial infarction; PAD, peripheral artery disease.

Table S2. Percent change in body weight and proportionate change in hsCRP by visit week on trial

Percent body weight loss (Multiple imputation using all available data and LOCF after deaths)					
	Semaglutide		Placebo		Treatment difference
Visit week	N	Mean (95% CI)	N	Mean (95% CI)	Mean (95% CI)
0	8803	0.00 (0.00, 0.00)	8801	0.00 (0.00, 0.00)	0.00 (0.00, 0.00)
4	8803	−1.38 (−1.42, −1.35)	8801	−0.27 (−0.31, −0.24)	−1.11 (−1.16, −1.06)
8	8803	−2.75 (−2.81, −2.70)	8801	−0.43 (−0.48, −0.39)	−2.32 (−2.39, −2.25)
12	8803	−4.04 (−4.11, −3.98)	8801	−0.57 (−0.62, −0.51)	−3.48 (−3.57, −3.39)
16	8803	−5.25 (−5.33, −5.17)	8801	−0.71 (−0.77, −0.64)	−4.54 (−4.65, −4.44)
20	8803	−6.40 (−6.50, −6.30)	8801	−0.83 (−0.90, −0.75)	−5.57 (−5.70, −5.45)
26	8803	−7.39 (−7.50, −7.28)	8801	−0.83 (−0.91, −0.75)	−6.56 (−6.70, −6.42)
39	8803	−8.54 (−8.68, −8.40)	8801	−0.84 (−0.93, −0.74)	−7.70 (−7.87, −7.53)
52	8803	−9.02 (−9.18, −8.86)	8801	−0.75 (−0.86, −0.65)	−8.27 (−8.45, −8.08)
65	8803	−9.19 (−9.36, −9.02)	8801	−0.73 (−0.84, −0.61)	−8.46 (−8.66, −8.26)
78	8803	−9.12 (−9.29, −8.94)	8801	−0.80 (−0.93, −0.68)	−8.31 (−8.53, −8.10)
91	8803	−9.11 (−9.29, −8.92)	8801	−0.91 (−1.04, −0.79)	−8.19 (−8.42, −7.97)
104	8803	−8.99 (−9.18, −8.80)	8801	−0.89 (−1.02, −0.75)	−8.10 (−8.33, −7.87)
117	8803	−9.02 (−9.21, −8.82)	8801	−0.95 (−1.09, −0.81)	−8.07 (−8.30, −7.83)
130	8803	−8.87 (−9.07, −8.68)	8801	−1.10 (−1.24, −0.95)	−7.78 (−8.02, −7.53)
143	8803	−8.81 (−9.02, −8.61)	8801	−1.19 (−1.34, −1.04)	−7.63 (−7.88, −7.37)
156	8803	−8.80 (−9.00, −8.59)	8801	−1.27 (−1.43, −1.11)	−7.53 (−7.79, −7.27)
169	8803	−8.85 (−9.06, −8.64)	8801	−1.39 (−1.56, −1.23)	−7.46 (−7.72, −7.19)
182	8803	−8.83 (−9.05, −8.62)	8801	−1.49 (−1.66, −1.32)	−7.34 (−7.62, −7.07)
195	8803	−8.91 (−9.14, −8.69)	8801	−1.51 (−1.69, −1.34)	−7.40 (−7.68, −7.12)
208	8803	−9.04 (−9.26, −8.81)	8801	−1.58 (−1.77, −1.39)	−7.46 (−7.75, −7.17)
221	8803	−9.48 (−9.70, −9.25)	8801	−1.60 (−1.79, −1.41)	−7.88 (−8.17, −7.58)

Percent change in GM hsCRP (Multiple imputation using all available data and LOCF after deaths)					
	Semaglutide		Placebo		Treatment difference
Visit week	N	GM (95% CI)	N	GM (95% CI)	GM (95% CI)
0	8798	0.0 (0.0, 0.0)	8795	0.0 (0.0, 0.0)	0.0 (0.0, 0.0)
4	8798	−11.0 (−13.1, −8.8)	8795	0.5 (−1.7, 2.8)	−11.4 (−14.3, −8.4)
8	8798	−17.2 (−19.1, −15.2)	8795	−2.0 (−4.3, 0.2)	−15.4 (−18.2, −12.6)
12	8798	−22.5 (−24.4, −20.6)	8795	−3.3 (−5.5, −1.1)	−19.9 (−22.5, −17.1)
16	8798	−26.1 (−27.9, −24.3)	8795	−2.3 (−4.6, −0.1)	−24.4 (−26.9, −21.7)
20	8798	−26.2 (−27.8, −24.7)	8795	−2.2 (−4.1, −0.3)	−24.6 (−26.7, −22.3)
52	8798	−38.6 (−40.0, −37.2)	8795	−3.9 (−5.8, −1.9)	−36.2 (−38.1, −34.2)
104	8798	−38.7 (−40.1, −37.2)	8795	−0.8 (−3.0, 1.4)	−38.2 (−40.2, −36.2)
156	8798	−37.2 (−38.8, −35.5)	8795	−3.0 (−5.3, −0.6)	−35.2 (−37.5, −32.9)
208	8798	−35.2 (−37.1, −33.3)	8795	−4.0 (−6.7, −1.3)	−32.5 (−35.2, −29.7)

Participants in the semaglutide arm were estimated to have lost 6.4% (95% CI 6.3, 6.5) and 9.0% (95% CI 8.8, 9.2) of body weight at 20 weeks and 104 weeks after randomization, respectively. For participants in the placebo arm, the corresponding estimates were 0.8% (95% CI 0.8%, 0.9%) and 0.9% (95% CI 0.8, 1.0) at 20 weeks and 104 weeks, resulting in a difference in average percent body weight loss between the treatment arms of 5.6% (95% CI 5.5, 5.7) at 20 weeks and 8.1% (95% CI 7.9, 8.3) (**Table S1**).

Discussion: A graphical description of the data in **Table S2** is presented in **Figure 1**, which provides the GM of the proportionate change in hsCRP and the average percent change in body weight as measured during the trial for each treatment arm. Note that the justification for using the same scale of y-axis in Figure 1 for both body weight and hsCRP is based on the approximate equivalence of HRs estimated from the results in **Table S4**. To further elaborate on this, the SELECT placebo data indicates a 10 kg difference in lower body weight at baseline which equaled a 10.34% difference from the mean baseline body weight; such a difference in baseline body weight was associated with an HR of 0.98 for MACE. Similarly, a 10.34% difference in baseline hsCRP in the placebo group would also estimate an HR of 0.98. Thus, use of the same y-axis in **Figure 1**, as supported by the data in **Table S2**, is a reasonable, appropriate representation of these changes.

GM, geometric mean; HR, hazard ratio; hsCRP, high-sensitivity C-reactive protein; LOCF, last observation carried forward; and MACE, major adverse cardiovascular events.

Table S3. Ratio-to-baseline hsCRP at week 104 by additional baseline subgroups

Subgroup at baseline	Ratio-to-baseline hsCRP		ETR (95% CI)	Interaction *P* value
	Semaglutide	Placebo		
**Age, years**				
<55	0.58 (n=2016)	1.01 (n=2059)	0.57 (0.54, 0.61)	0.0177
55–<65	0.61 (n=3295)	0.96 (n=3244)	0.64 (0.61, 0.67)	
65–<75	0.61 (n=2560)	0.97 (n=2596)	0.63 (0.59, 0.66)	
≥75	0.68 (n=666)	1.00 (n=627)	0.67 (0.60, 0.75)	
**Region**				
North America	0.62 (n=2118)	1.03 (n=2139)	0.61 (0.57, 0.64)	0.7765
Europe	0.59 (n=3253)	0.95 (n=3275)	0.63 (0.60, 0.66)	
Asia	0.58 (n=1084)	0.93 (n=1076)	0.62 (0.57, 0.68)	
Other	0.64 (n=2082)	1.01 (n=2036)	0.63 (0.59, 0.67)	
**Race**				
Asian	0.57 (n=707)	0.92 (n=707)	0.62 (0.56, 0.69)	0.9913
Black or African American	0.75 (n=328)	1.19 (n=312)	0.63 (0.53, 0.74)	
White	0.60 (n=7167)	0.97 (n=7178)	0.62 (0.60, 0.64)	
Other	0.73 (n=240)	1.21 (n=258)	0.61 (0.51, 0.72)	
**Ethnicity**				
Hispanic/Latino	0.62 (n=878)	1.01 (n=862)	0.62 (0.56, 0.68)	0.8875
Not Hispanic/Latino	0.61 (n=7564)	0.98 (n=7591)	0.62 (0.60, 0.64)	
**Heart failure**				
Yes	0.64 (n=2048)	1.00 (n=2020)	0.64 (0.60, 0.68)	0.3699
No	0.60 (n=6488)	0.97 (n=6505)	0.62 (0.60, 0.64)	
**BMI, kg/m^2**				
<30	0.59 (n=2465)	0.93 (n=2393)	0.63 (0.60, 0.67)	0.8317
30–<35	0.59 (n=3601)	0.95 (n=3679)	0.63 (0.60, 0.66)	
35–<40	0.65 (n=1634)	1.06 (n=1597)	0.61 (0.57, 0.65)	
40–<45	0.66 (n=558)	1.08 (n=572)	0.61 (0.54, 0.68)	
≥45	0.71 (n=279)	1.22 (n=285)	0.58 (0.49, 0.69)	
**eGFR, mL/min/1.73 m^2**				
<60	0.65 (n=920)	1.04 (n=870)	0.63 (0.57, 0.69)	0.8432
≥60	0.60 (n=7605)	0.97 (n=7645)	0.62 (0.60, 0.64)	
**KDIGO**				
1	0.60 (n=6558)	0.96 (n=6658)	0.62 (0.60, 0.64)	0.9712
2	0.64 (n=1318)	1.03 (n=1248)	0.63 (0.58, 0.68)	
3	0.65 (n=336)	1.07 (n=311)	0.61 (0.52, 0.71)	
4	0.72 (n=132)	1.11 (n=116)	0.65 (0.50, 0.84)	

Data are estimated geometric mean ratios to baseline and ETRs, analyzed using covariance with treatment group, subgroup, and treatment group-by-subgroup interaction as fixed factors, and baseline value as a covariate, based on data from the in-trial observation period. Multiple imputation via linear regression was used to impute missing week 104 data separately for each treatment group. The model included the baseline value as a covariate and was fitted to all patients with a measurement regardless of treatment status at week 104. Mean estimates were adjusted according to observed baseline distribution. Baseline hsCRP was log-transformed prior to analysis and the estimates back-transformed afterwards. (n) denotes the number of patients contributing to the analysis.
BMI, body mass index; eGFR, estimated glomerular filtration rate; ETR, estimated treatment ratio; hsCRP, high-sensitivity C-reactive protein; and KDIGO, Kidney Disease: Improving Global Outcomes.

Table S4. Changes from baseline to week 104 as associated with baseline measurements of body weight and hsCRP in all patients, and in male and female patients, in linear continuous models

**Percent body weight loss at week 104 by linear continuous model of baseline body weight** (Multiple imputation using all available data and LOCF after deaths)			
**Regression parameter**	**Semaglutide**<br>**Mean (95% CI)**	**Placebo**<br>**Mean (95% CI)**	**Semaglutide - placebo difference**<br>**Mean (95% CI)**
**All patients**	n=8803	n=8801	
Intercept: Percent change for 90 kg	−8.98 (−9.18, −8.78)	−0.85 (−0.99, −0.71)	−8.13 (−8.38, −7.88)
Slope: Diff in percent change per 5 kg higher baseline	0.00 (−0.06, 0.05)	−0.03 (−0.07, 0.01)	0.02 (−0.04, 0.09)
**Male patients**	n=6355	n=6377	
Intercept: Percent change for 90 kg	−7.53 (−7.75, −7.31)	−0.63 (−0.80, −0.46)	−6.90 (−7.18, −6.62)
Slope: Diff in percent change per 5 kg higher baseline	−0.13 (−0.18, −0.07)	−0.04 (−0.08, 0.00)	−0.09 (−0.16, −0.02)
**Female patients**	n=2448	n=2424	
Intercept: Percent change for 90 kg	−12.19 (−12.61, −11.76)	−1.39 (−1.68, −1.10)	−10.80 (−11.31, −10.28)
Slope: Diff in percent change per 5 kg higher baseline	−0.21 (−0.34, −0.09)	−0.09 (−0.18, −0.01)	−0.12 (-0.27, 0.03)

**Ratio of week 104 hsCRP to baseline by linear continuous model of baseline hsCRP** (Multiple imputation using all available data and LOCF after deaths)			
**Regression parameter**	**Semaglutide**<br>**GM (95% CI)**	**Placebo**<br>**GM (95% CI)**	**Semaglutide: placebo ratio**<br>**GM (95% CI)**
**All patients**	n=8798	n=8795	
Intercept: Ratio for 1 mg/dL	0.80 (0.78, 0.82)	1.27 (1.25, 1.30)	0.62 (0.60, 0.65)
Slope: Prop decr per 25% lower baseline	0.68 (0.66, 0.69)	0.68 (0.67, 0.69)	1.00 (0.97, 1.02)
**Male patients**	n=6351	n=6372	
Intercept: Ratio for 1 mg/dL	0.82 (0.79, 0.84)	1.25 (1.22, 1.29)	0.65 (0.63, 0.68)
Slope: Prop decr per 25% lower baseline	0.67 (0.66, 0.69)	0.67 (0.65, 0.68)	1.00 (0.97, 1.04)
**Female patients**	n=2447	n=2423	
Intercept: Ratio for 1 mg/dL	0.73 (0.69, 0.77)	1.34 (1.27, 1.41)	0.54 (0.51, 0.59)
Slope: Prop decr per 25% lower baseline	0.70 (0.68, 0.73)	0.69 (0.67, 0.72)	1.02 (0.97, 1.07)

Based on the slope estimates from linear regression models with baseline measurement as a linear continuous factor in the full analysis set, percent change in body weight (in the context of overweight/obesity as a SELECT entry criteria) was not associated with baseline body weight in either treatment arm. The difference in intercepts reflects the treatment effect of semaglutide on body weight change at week 104. However, when analyzed within subgroups by sex, small associations between the percent change in body weight at week 104 and baseline body weight are observed in each treatment arm; stronger associations are seen in the semaglutide arm than in the placebo arm for both male and female patients, with the association in

female patients numerically greater than that in male patients. From the intercept estimates within subgroups by sex, a larger treatment effect is estimated for female than for male patients.

Corresponding analyses based on linear regression models of the log ratio of week 104 hsCRP measurements to baseline show a strong linear association with log baseline hsCRP in each treatment group in the full analysis set as well as in each subgroup by sex. There is no evidence that the linear trend between the changes in hsCRP at week 104 and the baseline hsCRP differs between treatment arms. The difference in intercepts from the linear regression models reflects the treatment effect of semaglutide on hsCRP, which is similar between sexes.

Diff, difference; GM, geometric mean; hsCRP, high-sensitivity C-reactive protein; and LOCF, last observation carried forward.

Table S5. Joint distribution of incidence of MACE and baseline body weight or hsCRP with linear continuous modeling

Joint distribution of incidence of MACE and baseline body weight or hsCRP at baseline				
**Linear continuous modeling of risk of MACE per 5 kg lower baseline body weight**				
**Semaglutide**		**Placebo**		**Interaction**
HR (95% CI); *P* value	Quadratic term *P* value	HR (95% CI); *P* value	Quadratic term *P* value	Semaglutide: Placebo HR (95% CI); *P* value
All patients				
0.96 (0.94, 0.99); *P*=0.001	0.493	0.99 (0.97, 1.01); *P*=0.285	0.050	0.98 (0.95, 1.01); *P*=0.107
Male patients				
0.97 (0.95, 1.00); *P*=0.043	0.906	1.00 (0.97, 1.02); *P*=0.722	0.017	0.98 (0.94, 1.01); *P*=0.216
Female patients				
0.96 (0.91, 1.01); *P*=0.107	0.019	1.01 (0.97, 1.07); *P*=0.562	0.330	0.95 (0.88, 1.01); *P*=0.122
**Linear continuous modeling of risk of MACE per 25% difference in baseline hsCRP**				
**Semaglutide**		**Placebo**		**Interaction**
HR (95% CI); *P* value	Quadratic term *P* value	HR (95% CI); *P* value	Quadratic term *P* value	Semaglutide: Placebo HR (95% CI); *P* value
All patients				
0.92 (0.91, 0.94); *P*=0.000	0.648	0.94 (0.93, 0.96); *P*=0.000	0.468	0.98 (0.95, 1.03); *P*=0.122
Male patients				
0.91 (0.89, 0.93); *P*=0.000	0.313	0.93 (0.91, 0.95); *P*=0.000	0.863	0.98 (0.95, 1.03); *P*=0.179
Female patients				
0.94 (0.90, 0.99); *P*=0.009	0.320	0.96 (0.92, 1.00); *P*=0.050	0.245	0.98 (0.93, 1.06); *P*=0.617

Among the 8753 participants with available data who were randomized to placebo (n=8801), a first-order (linear) trend in an association between baseline hsCRP and on-trial risk of MACE was assessed using a Cox proportional hazards model with log-transformed baseline hsCRP modeled as a linear continuous covariate. From this analysis, a clinically important and statistically significant HR of 0.944 (95% CI 0.927, 0.962; *P*<0.0001) was estimated for placebo patients with baseline hsCRP levels 25% lower than a comparison placebo group (eg, 1.5 vs 2.0 mg/L or 3.0 vs 4.0 mg/L). This analysis estimates that patients on placebo with a baseline hsCRP of 0.47 mg/L (the 10th percentile of the enrolled population) would have a risk of on-trial MACE that was 44% lower (HR 0.56) than that for placebo patients with baseline hsCRP of 8.59 mg/L (the 90th percentile). Analyses exploring departures from straight-line associations found no evidence of a quadratic trend (*P*=0.46). A similar trend was found among the 8732 participants with available data who were randomized to the semaglutide arm (n=8803): an HR of 0.924 (95% CI 0.906, 0.943; *P*<0.0001) was estimated for semaglutide patients with baseline hsCRP levels 25% lower than a comparison semaglutide group, with no

evidence of a quadratic departure from a straight-line relationship (*P*=0.65). A test for an interaction between treatment and baseline hsCRP in the incidence of MACE was not statistically significant (*P*=0.12).

A corresponding analysis of the prognostic value of baseline body weight for on-trial incidence of MACE in placebo patients was not statistically significant: an HR of 0.989 (95% CI 0.969, 1.01; *P*=0.285) was estimated for placebo patients with baseline body weight 5 kg lower than a comparison placebo group. This analysis estimates that placebo patients with baseline body weight of 76.8 kg (the 10th percentile of the enrolled population) would have a risk of on-trial MACE that was a statistically nonsignificant 9% lower (HR 0.91) than that for placebo patients with baseline body weight of 119.6 kg (the 90th percentile). Analyses exploring departures from straight-line associations on the placebo arm found no strong evidence of a quadratic trend (*P*=0.05). Among participants randomized to the semaglutide arm, a statistically significant first-order trend toward lower risk of MACE in patients having lower baseline body weight was found: an HR of 0.965 (95% CI 0.944, 0.986; *P*=0.001) was estimated for semaglutide patients with baseline body weight 5 kg lower than a comparison semaglutide group. This analysis estimates that semaglutide patients with baseline body weight of 76.8 kg (the 10th percentile of the enrolled population) would have a risk of on-trial MACE that was a 27% lower (HR 0.73) than that for semaglutide patients with baseline body weight of 119.6 kg (the 90th percentile). There was no evidence of a quadratic departure from a straight-line relationship on the semaglutide arm (*P*=0.49). A test for an interaction between treatment and baseline body weight in the incidence of MACE was not statistically significant (*P*=0.11).

Discussion: In SELECT, baseline hsCRP levels were found to be prognostic of MACE risk in both treatment groups, and the slope of the first-order trend was similar across arms, suggesting that in the prognostic models for risk of MACE, the effects due to treatment and the effects due to baseline hsCRP levels were approximately additive on the log hazard scale. As such, further characterizing the plausibility that an achieved lower hsCRP level might be a marker of the treatment effect on incidence of MACE is of interest. In SELECT, treatment with semaglutide was associated with a 20% decrease in risk of MACE (HR 0.80). Using the prognostic effect of baseline hsCRP in the placebo group as a reference, an HR of 0.80 would be the difference in risk between two groups where one group had a 67.4% lower baseline hsCRP than the other. As shown in Figure 1, changes in hsCRP levels during SELECT plateaued by week 52, and at week 104, the semaglutide arm instead trended toward only 37.8% lower hsCRP levels relative to placebo. Such a difference would correspond to an HR of 0.91 between treatment arms if the resulting hsCRP levels followed the same prognostic pattern as baseline hsCRP levels in the placebo group. **Hence, while these analyses support the potential plausibility of achieved hsCRP levels as a useful marker of *some* amount of decreased risk of MACE due to semaglutide, it is *not* plausible that semaglutide's effect on absolute hsCRP levels will capture the total treatment effect.** Of note, changes in hsCRP in response to semaglutide may involve effects beyond the absolute measurements, as explored in subsequent analyses.

Baseline body weight was not strongly prognostic of MACE risk in the placebo group. Using the prognostic effect of baseline body weight in the placebo group as a reference, the observed treatment effect of an HR of 0.80 on risk of MACE would be the difference in risk between two groups where one group had 98.8 kg lower baseline body weight than the other – a difference that must be judged in relation to the mean baseline body weight of 96.7 kg for SELECT patients. Changes in body weight during SELECT plateaued by week 52, and at week 104, the semaglutide arm instead averaged 8.23 kg greater body weight loss than the placebo group. Such a difference would correspond to an HR of 0.98 between treatment arms if the resulting body weight followed the same prognostic pattern as baseline body weight in the placebo group. Thus, these analyses do not support the plausibility of achieved weight as a useful marker of decreased risk of MACE due to semaglutide. However, it is again noteworthy that changes in body weight from semaglutide may represent other effects beyond the absolute body weight measurements, as noted in the manuscript and explored in subsequent analyses.

Of note, the effect of baseline body weight on risk of MACE was more prognostic in the semaglutide arm than in the placebo arm. While no statistically significant interaction between treatment and baseline body weight was observed, it should be recognized that the statistical precision to detect trends within a treatment arm is greater than the statistical precision with which interactions can be quantified. For instance, if a clinical trial

affords 90% statistical power to detect a specified first-order trend in risk within a single arm, only an approximately 63% power exists to detect that such a trend differs from that observed in an equal-sized control group that truly has no such association.

Among placebo patients, linear continuous modeling shows baseline hsCRP is highly prognostic of on-trial risk of MACE, with similar estimates of associations for all patients, and male or female patients, and with no strong evidence of nonlinear trends. The linear continuous modeling of risk of MACE by baseline body weight shows no clear first-order trend in prognostic effect, albeit with some weak evidence of a nonlinear quadratic trend in males.

Among semaglutide patients, linear continuous modeling shows baseline hsCRP is again highly prognostic of on-trial risk of MACE, with estimates of associations similar across treatment arms for all patients, and male or female patients, without suggestion of nonlinear quadratic trends. Estimated trends in prognostic effect of baseline hsCRP were slightly stronger for male patients than female patients.

Results for the semaglutide arm did find a first-order trend toward lower risk of MACE with lower baseline body weight among all patients and without suggestion of nonlinear quadratic effects among male patients. Among female patients in the semaglutide arm, estimates of the first-order trend in risk of MACE were numerically similar, with some weak evidence of a nonlinear quadratic trend in female patients.

HR, hazard ratio; hsCRP, high-sensitivity C-reactive protein; and MACE, major adverse cardiovascular events.

Table S6. Correlations among observed changes in body weight and log hsCRP measurements while on trial at weeks 104 and 208

Correlations among changes in observed measurements on trial (no imputation)										
		Semaglutide				Placebo				
		Percent change in body weight		Change in log hsCRP		Percent change in body weight		Change in log hsCRP		
	Visit week	104	208	104	208	104	208	104	208
Percent change in body weight	104	1.000	0.801	0.266	0.229	1.000	0.627	0.080	0.035
	208	0.801	1.000	0.158	0.250	0.627	1.000	0.001	0.062
Change in log hsCRP	104	0.266	0.158	1.000	0.540	0.080	0.001	1.000	0.536
	208	0.229	0.250	0.540	1.000	0.035	0.062	0.536	1.000

Among patients in the placebo arm, the correlation between week 104 and week 208 measurements of percent change in body weight from baseline is moderately high (0.627), as is the correlation between week 104 and week 208 ratio of hsCRP measurements to baseline (0.536 as computed on log-transformed hsCRP). However, in the placebo arm, the percent change in body weight from baseline and the ratio of postrandomization hsCRP to baseline are not highly correlated to each other (0.080 at week 104 and 0.062 at week 208).

Among patients in the semaglutide arm, all measurements are significantly more highly correlated with each other than they were in the placebo arm ($P<0.0001$). Of particular note is the higher correlation between the percent change in body weight from baseline and the ratio of postrandomization hsCRP to baseline (0.266 at week 104 and 0.250 at week 208).

Discussion: It is not possible from these analyses to fully differentiate alternative hypotheses regarding the higher correlations between hsCRP and body weight among the semaglutide patients. These results are potentially consistent both with a hypothesized semaglutide effect on a common pathway that affects both body weight loss and inflammation and with a hypothesized semaglutide effect on separate pathways involving body weight loss and inflammation, where higher correlation between measures of body weight loss and hsCRP might reflect adherence to treatment.

hsCRP, high-sensitivity C-reactive protein.

Table S7. Joint distribution of percent change in body weight from baseline to week 104 and the ratio of week 104 hsCRP to baseline hsCRP

A. All patients: Joint distribution of body weight loss and change in hsCRP at week 104					
**Percent body weight change at week 104 by categories of decrease in hsCRP at week 104** (Multiple imputation using all available data and LOCF after deaths)					
	**Semaglutide**		**Placebo**		**Treatment difference**
**Percent change in hsCRP**	N	Percent change body weight Mean (95% CI)	N	Percent change body weight Mean (95% CI)	Semaglutide - Placebo mean diff (95% CI)
All	8803 (100%)	−8.99 (−9.18, −8.80)	8801 (100%)	−0.89 (−1.02, −0.76)	−8.10 (−8.32, −7.88)
hsCRP decr ≥50%	3596 (41%)	−11.60 (−11.91, −11.29)	1777 (20%)	−1.97 (−2.30, −1.63)	−9.63 (−10.06, −9.20)
50% > hsCRP decr ≥25%	1618 (18%)	−8.49 (−8.90, −8.08)	1423 (16%)	−1.38 (−1.71, −1.04)	−7.11 (−7.60, −6.62)
25% > hsCRP decr ≥0%	1054 (12%)	−7.10 (−7.60, −6.59)	1287 (15%)	−0.84 (−1.16, −0.51)	−6.26 (−6.82, −5.70)
hsCRP increase	2530 (29%)	−6.39 (−6.73, −6.05)	4307 (49%)	−0.30 (−0.49, −0.11)	−6.09 (−6.46, −5.72)
**Linear continuous modeling of percent body weight loss per 25% decrease in hsCRP at week 104**					
	**Semaglutide**		**Placebo**		**Interaction**
	Percent change weight Mean (95% CI); *P* value	Quadratic term *P* value	Percent change weight Mean (95% CI); *P* value	Quadratic term *P* value	Semaglutide - Placebo Mean diff (95% CI); *P* value
	−0.53 (−0.58, −0.48); *P*=0.000	0.0000	−0.12 (−0.16, −0.08); *P*=0.000	0.0000	−0.40 (−0.47, −0.34); *P*=0.000
**Ratio week 104 to baseline hsCRP by categories of percent body weight loss at week 104** (Multiple imputation using all available data and LOCF after deaths)					
	**Semaglutide**		**Placebo**		**Treatment difference**
**Percent weight loss**	N	Ratio week 104 to baseline hsCRP GM (95% CI)	N	Ratio week 104 to baseline hsCRP GM (95% CI)	Semaglutide: Placebo HR (95% CI);
All	8798 (100%)	0.61 (0.60, 0.63)	8795 (100%)	0.99 (0.97, 1.01)	0.62 (0.60, 0.64)
Weight loss ≥10%	3757 (43%)	0.48 (0.46, 0.50)	630 (7%)	0.84 (0.76, 0.92)	0.57 (0.52, 0.63)
5% ≤ weight loss <10%	2039 (23%)	0.66 (0.63, 0.69)	1285 (15%)	0.94 (0.88, 1.00)	0.71 (0.66, 0.76)
0% ≤ weight loss <5%	1790 (20%)	0.75 (0.71, 0.79)	2896 (33%)	0.97 (0.93, 1.01)	0.77 (0.73, 0.82)
Weight gain	1212 (14%)	0.85 (0.80, 0.90)	3984 (45%)	1.06 (1.02, 1.09)	0.80 (0.75, 0.86)
**Linear continuous modeling of percent change in hsCRP per 5% decrease in body weight at week 104**					
	**Semaglutide**		**Placebo**		**Interaction**
	Ratio week 104 to baseline hsCRP GM (95% CI); *P* value	Quadratic term *P* value	Ratio week 104 to baseline hsCRP GM (95% CI); *P* value	Quadratic term *P* value	Semaglutide est GM: Placebo est GM GM ratio (95% CI); *P* value
	0.87 (0.86, 0.88); *P*=0.000	0.0924	0.94 (0.93, 0.96); *P*=0.000	0.5649	0.92 (0.90, 0.94); *P*=0.000

B. Male patients: Joint distribution of body weight loss and change in hsCRP at week 104

Percent body weight change at week 104 by categories of decrease in hsCRP at week 104
(Multiple imputation using all available data and LOCF after deaths)

Percent change in hsCRP	Semaglutide N	Semaglutide Percent change body weight Mean (95% CI)	Placebo N	Placebo Percent change body weight Mean (95% CI)	Treatment difference Semaglutide - Placebo mean diff (95% CI)
All	6355 (100%)	−7.76 (−7.96, −7.57)	6377 (100%)	−0.70 (−0.85, −0.56)	−7.06 (−7.30, −6.82)
hsCRP decr ≥50%	2420 (38%)	−9.68 (−10.00, −9.35)	1255 (20%)	−1.55 (−1.92, −1.18)	−8.13 (−8.59, −7.66)
50% > hsCRP decr ≥25%	1182 (19%)	−7.66 (−8.09, −7.22)	1033 (16%)	−1.11 (−1.49, −0.74)	−6.55 (−7.08, −6.01)
25% > hsCRP decr ≥0%	776 (12%)	−6.66 (−7.20, −6.11)	914 (14%)	−0.75 (−1.11, −0.38)	−5.91 (−6.52, −5.30)
hsCRP increase	1972 (31%)	−5.92 (−6.27, −5.56)	3170 (50%)	−0.22 (−0.43, −0.01)	−5.70 (−6.09, −5.30)

Linear continuous modeling of percent body weight loss per 25% decrease in hsCRP at week 104

	Semaglutide Percent change weight Mean (95% CI); *P* value	Semaglutide Quadratic term *P* value	Placebo Percent change weight Mean (95% CI); *P* value	Placebo Quadratic term *P* value	Interaction Semaglutide est mean - Placebo est mean Mean diff (95% CI); *P* value
	−0.36 (−0.42, −0.31); *P*=0.000	0.0436	−0.08 (−0.12, −0.03); *P*=0.000	0.0000	−0.29 (−0.35, −0.22); *P*=0.000

Ratio week 104 to baseline hsCRP by categories of percent body weight loss at week 104
(Multiple imputation using all available data and LOCF after deaths)

Percent body weight loss	Semaglutide N	Semaglutide Ratio week 104 to baseline hsCRP Mean (95% CI)	Placebo N	Placebo Ratio week 104 to baseline hsCRP Mean (95% CI)	Treatment difference Semaglutide: Placebo HR (95% CI)
All	6351 (100%)	0.65 (0.64, 0.67)	6372 (100%)	1.01 (0.99, 1.04)	0.64 (0.62, 0.67)
Weight loss ≥10%	2390 (38%)	0.52 (0.50, 0.55)	375 (6%)	0.93 (0.81, 1.06)	0.56 (0.49, 0.64)
5% ≤ weight loss <10%	1581 (25%)	0.67 (0.64, 0.71)	873 (14%)	0.97 (0.89, 1.05)	0.70 (0.64, 0.76)
0% ≤ weight loss <5%	1447 (23%)	0.77 (0.73, 0.82)	2164 (34%)	1.00 (0.95, 1.04)	0.77 (0.72, 0.83)
Weight gain	933 (15%)	0.86 (0.80, 0.92)	2959 (46%)	1.06 (1.02, 1.10)	0.81 (0.76, 0.88)

Linear continuous modeling of percent change in hsCRP per 5% decrease in body weight at week 104

	Semaglutide Ratio week 104 to baseline hsCRP GM (95% CI); *P* value	Semaglutide Quadratic term *P* value	Placebo Ratio week 104 to baseline hsCRP GM (95% CI); *P* value	Placebo Quadratic term *P* value	Interaction Semaglutide est GM: Placebo est GM GM ratio (95% CI); *P* value
	0.88 (0.87, 0.90); *P*=0.000	0.7233	0.96 (0.94, 0.98); *P*=0.000	0.4280	0.92 (0.89, 0.95); *P*=0.000

C. Female patients: Joint distribution of body weight loss and change in hsCRP at week 104					
**Percent body weight change at week 104 by categories of decrease in hsCRP at week 104** (Multiple imputation using all available data and LOCF after deaths)					
	**Semaglutide**		**Placebo**		**Treatment difference**
**Percent change in hsCRP**	N	Percent change body weight Mean (95% CI)	N	Percent change body weight Mean (95% CI)	Semaglutide - Placebo mean diff (95% CI)
All	2448 (100%)	−12.17 (−12.60, −11.75)	2424 (100%)	−1.38 (−1.67, −1.09)	−10.79 (−11.29, −10.29)
hsCRP decr ≥50%	1174 (48%)	−15.58 (−16.20, −14.96)	523 (22%)	−2.96 (−3.67, −2.25)	−12.62 (−13.50, −11.74)
50% > hsCRP decr ≥25%	435 (18%)	−10.73 (−11.64, −9.83)	390 (16%)	−2.07 (−2.78, −1.37)	−8.66 (−9.72, −7.60)
25% > hsCRP decr ≥0%	278 (11%)	−8.31 (−9.48, −7.15)	374 (15%)	−1.05 (−1.75, −0.35)	−7.26 (−8.53, −6.00)
hsCRP increase	560 (23%)	−8.07 (−8.98, −7.16)	1136 (47%)	−0.52 (−0.95, −0.10)	−7.55 (−8.49, −6.60)
**Linear continuous modeling of percent body weight loss per 25% decrease in hsCRP at week 104**					
	**Semaglutide**		**Placebo**		**Interaction**
	Percent change weight Mean (95% CI); *P* value	Quadratic term *P* value	Percent change weight Mean (95% CI); *P* value	Quadratic term *P* value	Semaglutide est mean - Placebo est mean Mean diff (95% CI); *P* value
	−0.80 (−0.92, −0.69); *P*=0.000	0.0014	−0.24 (−0.33, −0.16); *P*=0.000	0.2793	−0.56 (−0.70, −0.41); *P*=0.000
**Ratio week 104 to baseline hsCRP by categories of percent body weight loss at week 104**					
	**Semaglutide**		**Placebo**		**Treatment difference**
**Percent body weight loss**	N	Ratio week 104 to baseline hsCRP Mean (95% CI)	N	Ratio week 104 to baseline hsCRP Mean (95% CI)	Semaglutide: Placebo HR (95% CI)
All	2447 (100%)	0.52 (0.50, 0.54)	2423 (100%)	0.93 (0.90, 0.97)	0.56 (0.52, 0.59)
Weight loss ≥10%	1366 (56%)	0.42 (0.39, 0.44)	255 (11%)	0.73 (0.63, 0.84)	0.57 (0.49, 0.67)
5% ≤ weight loss <10%	458 (19%)	0.62 (0.56, 0.69)	412 (17%)	0.87 (0.79, 0.97)	0.71 (0.62, 0.81)
0% ≤ weight loss <5%	343 (14%)	0.67 (0.60, 0.75)	732 (30%)	0.89 (0.83, 0.96)	0.76 (0.67, 0.85)
Weight gain	279 (11%)	0.81 (0.72, 0.93)	1025 (42%)	1.06 (0.99, 1.12)	0.77 (0.68, 0.88)
**Linear continuous modeling of percent change in hsCRP per 5% decrease in body weight at week 104**					
	**Semaglutide**		**Placebo**		**Interaction**
	Ratio week 104 to baseline hsCRP GM (95% CI); *P* value	Quadratic term *P* value	Ratio week 104 to baseline hsCRP GM (95% CI); *P* value	Quadratic term *P* value	Semaglutide est GM: Placebo est GM GM ratio (95% CI); *P* value
	0.87 (0.85, 0.88); *P*=0.000	0.3917	0.92 (0.89, 0.95); *P*=0.000	0.1605	0.94 (0.91, 0.98); *P*=0.001

The tables describing the joint distribution of week 104 changes from baseline in body weight and changes in hsCRP for all patients show clear trends toward patients on semaglutide having a greater tendency for reduction in body weight and reduction in hsCRP than placebo patients, with differences between the treatment arms being greatest for strata having the largest body weight loss or the largest decrease in hsCRP. Both linear continuous modeling of percent body weight loss on changes in hsCRP and linear continuous modeling of changes in hsCRP on percent body weight loss show highly significant first-order trends in both treatment arms, but with greater trends among semaglutide patients than placebo patients. First-order trend in percent change in body weight per 25% decrease in hsCRP is -0.53 for semaglutide patients and -0.12 for placebo patients, and first-order trend in proportionate change in hsCRP per 5% decrease in body weight loss is 0.87 for semaglutide patients and 0.94 for placebo patients.

No strong evidence exists for a nonlinear quadratic association on either treatment arm when modeling change in hsCRP as a function of change in body weight. However, there was strong evidence of a nonlinear quadratic association when modeling change in body weight as a function of change in hsCRP. Such departures from straight-line models are consistent with patterns of body weight loss associated with late-stage chronic disease (but not proof of their presence).

Results of corresponding analyses within subgroups defined by sex showed the same general trends as those in all patients.

decr, decrease; Diff, difference; est, estimated; GM, geometric mean; hsCRP, high-sensitivity C-reactive protein; and LOCF, last observation carried forward.

Table S8. On-treatment ratio-to-baseline hsCRP by change in body weight of <2% versus ≥2% at week 104 (first on-treatment period)

Body weight loss	Ratio-to-baseline hsCRP		ETR (95% CI)	Interaction *P* value
	Semaglutide	Placebo		
<2%	0.83 (n=803)	1.02 (n=4017)	0.81 (0.75, 0.87)	<0.001
≥2%	0.54 (n=5128)	0.93 (n=2566)	0.58 (0.56, 0.61)	

Data are estimated geometric mean ratios to baseline, analyzed using a mixed model for repeated measurements with treatment group, subgroup, and treatment group-by-subgroup interaction as fixed factors, and baseline value as a covariate, all nested within visit, using data from the first on-treatment observation period. (n) denotes the number of patients contributing to the analysis.
ETR, estimated treatment ratio; and hsCRP, high-sensitivity C-reactive protein.

Table S9. Joint linear continuous modeling of risk of MACE by week 104 change in body weight and baseline body weight or by week 104 change in hsCRP and baseline hsCRP

**Joint linear continuous modeling of risk of MACE per baseline body weight (5 kg difference) and percent body weight change at week 104 to baseline per 5% body weight change for all patients, and male and female patients (MACE events: all in trial; MI: all available data MAR; non-CV deaths; LOCF)**					
**All patients**					
	**Semaglutide (n=8803)**		**Placebo (n=8801)**		**Interaction**
	HR (95% CI); *P* value	Quadratic term *P* value	HR (95% CI); *P* value	Quadratic term *P* value	Semaglutide: Placebo HR (95% CI); *P* value
Baseline weight (per 5 kg diff)	0.96 (0.94, 0.99); *P*=0.001	0.448	0.99 (0.97, 1.01); *P*=0.301	0.043	0.98 (0.95, 1.00); *P*=0.101
Percent weight change week 104 to baseline (per 5% weight change)	**0.94 (0.90, 0.99); *P*=0.022**	**0.813**	**1.04 (0.98, 1.11); *P*=0.202**	**0.411 but not placebo**	0.91 (0.84, 0.98); *P*=0.015
**Male patients**					
	**Semaglutide (n=6355)**		**Placebo (n=6377)**		**Interaction**
	HR (95% CI); *P* value	Quadratic term *P* value	HR (95% CI); P value	Quadratic term *P* value	Semaglutide: Placebo HR (95% CI); *P* value
Baseline weight (per 5 kg diff)	0.97 (0.95, 1.00); *P*=0.041	0.906	1.00 (0.97, 1.02); *P*=0.778	0.010	0.98 (0.94, 1.01); *P*=0.191
Percent weight change week 104 to baseline (per 5% weight change)	0.98 (0.92, 1.05); *P*=0.634	0.203	1.08 (1.01, 1.17); *P*=0.034	0.542	0.91 (0.82, 1.00); *P*=0.055
**Female patients**					
	**Semaglutide (n=2448)**		**Placebo (n= 2424)**		**Interaction**
	HR (95% CI); *P* value	Quadratic term *P* value	HR (95% CI); *P* value	Quadratic term *P* value	Semaglutide: Placebo HR (95% CI); *P* value
Baseline weight (per 5 kg diff)	0.96 (0.91, 1.00); *P*=0.077	0.009	1.01 (0.97, 1.07); *P*=0.583	0.332	0.94 (0.88, 1.01); *P*=0.103
Percent weight change week 104 to baseline (per 5% weight change)	0.90 (0.82, 0.98); *P*=0.015	0.807	0.97 (0.86, 1.09); *P*=0.586	0.221	0.93 (0.80, 1.08); *P*=0.319

Joint linear continuous modeling of risk of MACE per 25% decrease in hsCRP at week 104 and baseline hsCRP (MACE events: all in trial; MI: all available data MAR; non-CV deaths; LOCF)					
**All patients**					
	**Semaglutide (n=8803)**		**Placebo (n=8801)**		**Interaction**
	HR (95% CI); *P* value	Quadratic term *P* value	HR (95% CI); *P* value	Quadratic term *P* value	Semaglutide: Placebo HR (95% CI); *P* value
Baseline hsCRP (per 25% decrease)	0.90 (0.88, 0.92); *P*=0.000	0.078	0.93 (0.91, 0.95); *P*=0.000	0.239	0.96 (0.93, 0.99); *P*=0.022
hsCRP ratio week 104 to baseline (per 25% decrease)	0.93 (0.91, 0.95); *P*=0.000	0.110	0.96 (0.94, 0.98); *P*=0.001	0.734	0.97 (0.93, 1.00); *P*=0.048
**Male patients**					
	**Semaglutide (n=6355)**		**Placebo (n=6377)**		**Interaction**
	HR (95% CI); *P* value	Quadratic term *P* value	HR (95% CI); *P* value	Quadratic term *P* value	Semaglutide: Placebo HR (95% CI); *P* value
Baseline hsCRP (per 25% decrease)	0.88 (0.86, 0.91); *P*=0.000	0.014	0.91 (0.89, 0.93); *P*=0.000	0.335	0.97 (0.94, 1.01); *P*=0.104
hsCRP ratio week 104 to baseline (per 25% decrease)	0.93 (0.91, 0.96); *P*=0.000	0.093	0.95 (0.92, 0.97); *P*=0.000	0.408	0.98 (0.95, 1.02); *P*=0.340
**Female patients**					
	**Semaglutide (n=2448)**		**Placebo (n=2424)**		**Interaction**
	HR (95% CI); *P* value	Quadratic term *P* value	HR (95% CI); *P* value	Quadratic term *P* value	Semaglutide: Placebo HR (95% CI); *P* value
Baseline hsCRP (per 25% decrease)	0.91 (0.87, 0.96); *P*=0.000	0.567	0.96 (0.91, 1.00); *P*=0.069	0.222	0.96 (0.89, 1.02); *P*=0.196
hsCRP ratio week 104 to baseline (per 25% decrease)	0.92 (0.87, 0.97); *P*=0.001	0.489	0.99 (0.94, 1.05); *P*=0.857	0.808	0.92 (0.86, 1.00); *P*=0.045

Inference on first-order trends in risk of MACE are provided by changes at week 104 in each marker (body weight, hsCRP) using joint linear continuous modeling of baseline and change in measurements in a proportional hazards regression within each treatment arm, along with inference about interactions between treatment and baseline or treatment and changes in markers. Also presented are the results for tests of nonlinear quadratic trends as a test for any evidence that the straight-line model used to describe first-order trends might not fully reflect data trends. Estimates are provided for the entire SELECT population as well as within each sex separately.

Discussion: Among placebo patients, no first-order trend exists toward a lower risk of MACE with greater body weight loss at week 104. Among semaglutide patients, both baseline body weight and weight loss at week 104 show evidence of first-order trends toward lower risk of MACE as body weight decreased on trial.

There was no strong evidence for a nonlinear quadratic trend for either body weight measure. The difference between semaglutide and placebo arms with respect to a linear trend in risk of MACE by week 104 body weight loss is nominally statistically significant. Within the strata defined by sex, estimates of the association between risk of MACE and either body weight loss at week 104 or baseline body weight are generally similar to that observed in the full analysis set.

In the full analysis set for each treatment arm, strong evidence exists that risk of MACE is associated with both proportionate change from baseline in hsCRP at week 104 and baseline hsCRP. The association between risk of MACE and each of the modeled covariates is stronger in the semaglutide group as compared to the placebo group. There is no evidence of a nonlinear quadratic departure from a straight-line relationship. In female patients, the associations between risk of MACE and either change in hsCRP or baseline hsCRP are numerically weaker than in male patients.

CV, cardiovascular; HR, hazard ratio; hsCRP, high-sensitivity C-reactive protein;
LOCF, last observation carried forward; MACE, major adverse cardiovascular events; MAR, missing at random; and MI, myocardial infarction.

Table S10. Joint distribution of on-trial MACE and change in body weight or hsCRP at week 104: Incidence rates by treatment arm within categories

Incidence of MACE by categories of percent change in body weight at week 104 (MACE events: all in trial; MI: all available data MAR; non-CV deaths; LOCF)							
**All patients**							
**Percent body weight loss at week 104**		**Semaglutide** Events / HPY	IR (95% CI)		**Placebo** Events / HPY	IR (95% CI)	Semaglutide: Placebo HR (95% CI)
All	n=8803 (100%)	569 / 286.5	1.99 (1.82, 2.15)	n=8801 (100%)	701 / 283.0	2.48 (2.29, 2.66)	0.80 (0.72, 0.90)
Weight loss ≥10%	n=3758 (43%)	222 / 122.8	**1.81 (1.57, 2.06)**	n=630 (7%)	62 / 19.5	**3.18 (2.35, 4.00)**	0.57 (0.42, 0.76)
5% ≤ weight loss <10%	n=2040 (23%)	139 / 66.5	**2.08 (1.72, 2.45)**	n=1286 (15%)	104 / 40.6	**2.56 (2.04, 3.08)**	0.81 (0.62, 1.07)
0% ≤ weight loss <5%	n=1791 (20%)	115 / 57.9	**1.98 (1.60, 2.37)**	n=2898 (33%)	234 / 92.6	**2.53 (2.19, 2.87)**	0.78 (0.62, 0.99)
Weight gain	n=1214 (14%)	93 / 39.3	**2.37 (1.86, 2.88)**	n=3986 (45%)	301 / 130.2	**2.31 (2.04, 2.58)**	1.03 (0.80, 1.31)
**Male patients**							
**Percent body weight loss at week 104**		**Semaglutide** Events / HPY	IR (95% CI)		**Placebo** Events / HPY	IR (95% CI)	Semaglutide: Placebo HR (95% CI)
All	n=6355 (100%)	443 / 207.0	2.14 (1.94, 2.34)	n=6377 (100%)	554 / 204.7	2.71 (2.48, 2.93)	0.79 (0.70, 0.90)
Weight loss ≥10%	n=2391 (38%)	164 / 77.9	2.11 (1.78, 2.44)	n=375 (6%)	45 / 11.6	3.88 (2.69, 5.06)	0.54 (0.38, 0.76)
5% ≤ weight loss <10%	n=1582 (25%)	107 / 51.8	2.07 (1.66, 2.48)	n=874 (14%)	81 / 27.7	2.91 (2.23, 3.59)	0.71 (0.52, 0.96)
0% ≤ weight loss <5%	n=1448 (23%)	95 / 47.0	2.03 (1.59, 2.47)	n=2166 (34%)	194 / 68.9	2.81 (2.39, 3.23)	0.72 (0.56, 0.94)
Weight gain	n=934 (15%)	76 / 30.4	2.51 (1.92, 3.11)	n=2962 (46%)	235 / 96.5	2.43 (2.11, 2.75)	1.03 (0.79, 1.36)
**Female patients**							
**Percent body weight loss at week 104**		**Semaglutide** Events / HPY	IR (95% CI)		**Placebo** Events / HPY	IR (95% CI)	Semaglutide: Placebo HR (95% CI)
All	n=2448 (100%)	126 / 79.5	1.58 (1.31, 1.86)	n=2424 (100%)	147 / 78.3	1.88 (1.57, 2.18)	0.84 (0.67, 1.07)
Weight loss ≥10%	n=1367 (56%)	58 / 44.9	1.30 (0.95, 1.64)	n=255 (11%)	17 / 8.0	2.17 (1.10, 3.23)	0.59 (0.34, 1.04)
5% ≤ weight loss <10%	n=458 (19%)	31 / 14.8	2.12 (1.33, 2.92)	n=412 (17%)	23 / 12.9	1.79 (1.00, 2.58)	1.19 (0.67, 2.12)
0% ≤ weight loss <5%	n=344 (14%)	20 / 11.0	1.79 (0.93, 2.65)	n=732 (30%)	41 / 23.7	1.72 (1.15, 2.29)	1.03 (0.58, 1.86)
Weight gain	n=279 (11%)	17 / 8.9	1.88 (0.92, 2.84)	n=1025 (42%)	66 / 33.7	1.95 (1.45, 2.45)	0.95 (0.54, 1.69)

Incidence of MACE by categories of change in hsCRP at week 104
(MACE events: all in trial; MI: all available data MAR; non-CV deaths; LOCF)

All patients

Ratio of week 104 hsCRP to baseline	Semaglutide			Placebo			Semaglutide: Placebo
		Events / HPY	IR (95% CI)		Events / HPY	IR (95% CI)	HR (95% CI)
All	n=8803 (100%)	569 / 286.5	**1.99 (1.82, 2.15)**	n=8801 (100%)	701 / 283.0	**2.48 (2.29, 2.66)**	0.80 (0.72, 0.90)
hsCRP decr ≥50%	n=3596 (41%)	199 / 118.2	**1.68 (1.44, 1.93)**	n=1777 (20%)	150 / 56.2	**2.67 (2.21, 3.13)**	0.63 (0.50, 0.79)
50% > hsCRP decr ≥25%	n=1618 (18%)	111 / 52.8	**2.10 (1.68, 2.51)**	n=1423 (16%)	96 / 46.0	**2.09 (1.64, 2.54)**	1.00 (0.75, 1.35)
25% > hsCRP decr ≥0%	n=1054 (12%)	71 / 34.5	**2.05 (1.54, 2.56)**	n=1287 (15%)	96 / 41.8	**2.30 (1.81, 2.79)**	0.89 (0.64, 1.24)
hsCRP increase	n=2530 (29%)	187 / 81.0	**2.31 (1.96, 2.65)**	n=4307 (49%)	358 / 138.8	**2.58 (2.30, 2.85)**	0.90 (0.75, 1.08)

Male patients

Ratio of week 104 hsCRP to baseline	Semaglutide			Placebo			Semaglutide: Placebo
		Events / HPY	IR (95% CI)		Events / HPY	IR (95% CI)	HR (95% CI)
All	n=6355 (100%)	443 / 207.0	2.14 (1.94, 2.34)	n=6377 (100%)	554 / 204.7	2.71 (2.48,2.93)	0.79 (0.70, 0.90)
hsCRP decr ≥50%	n=2420 (38%)	148 / 79.6	1.86 (1.55, 2.18)	n=1255 (20%)	115 / 39.9	2.89 (2.33,3.45)	0.65 (0.50, 0.84)
50% > hsCRP decr ≥25%	n=1182 (19%)	84 / 38.6	2.18 (1.69, 2.68)	n=1033 (16%)	74 / 33.4	2.23 (1.68,2.77)	0.98 (0.70, 1.37)
25% > hsCRP decr ≥0%	n=776 (12%)	56 / 25.7	2.19 (1.58, 2.81)	n=914 (14%)	68 / 29.7	2.28 (1.70,2.87)	0.96 (0.66, 1.41)
hsCRP increase	n=1972 (31%)	152 / 63.0	2.41 (2.01, 2.81)	n=3170 (50%)	297 / 101.6	2.92 (2.58,3.26)	0.83 (0.67, 1.02)

Female patients

Ratio of week 104 hsCRP to baseline	Semaglutide			Placebo			Semaglutide: Placebo
		Events / HPY	IR (95% CI)		Events / HPY	IR (95% CI)	HR (95% CI)
All	n=2448 (100%)	126 / 79.5	1.58 (1.31, 1.86)	n= 2424 (100%)	147 / 78.3	1.88 (1.57, 2.18)	0.84 (0.67, 1.07)
hsCRP decr ≥50%	n=1174 (48%)	50 / 38.5	1.30 (0.92, 1.68)	n=523 (22%)	35 / 16.3	2.12 (1.35, 2.88)	0.61 (0.39, 0.98)
50% > hsCRP decr ≥25%	n=435 (18%)	26 / 14.1	1.87 (1.12, 2.63)	n=390 (16%)	22 / 12.7	1.73 (0.94, 2.52)	1.09 (0.59, 2.01)
25% > hsCRP decr ≥0%	n=278 (11%)	14 / 8.8	1.60 (0.71, 2.49)	n=374 (15%)	28 / 12.1	2.34 (1.42, 3.25)	0.68 (0.35, 1.34)

hsCRP increase	n=560 (23%)	35 / 18.0	1.96 (1.28, 2.64)	n=1136 (47%)	61 / 37.2	1.65 (1.21, 2.08)	1.19 (0.77, 1.84)

IRs for on-trial MACE are presented within four categories of change (first column) in markers at week 104 for the percent change in body weight from baseline (upper table) or ratio-to-baseline hsCRP (lower table). Within each category, the HR between treatment groups is also presented. For each table, estimates are provided for the entire SELECT population as well as within each sex.

Discussion: Changes in body weight and MACE are provided here as context for hsCRP analyses. As previously reported, categorizing SELECT patients by treatment arm and body weight loss (week 104) revealed that the small subgroup of placebo patients losing more than 10% of baseline body weight had the highest IR for MACE (Deanfield J, Lincoff A, Kahn S et al. Semaglutide and cardiovascular outcomes by baseline and changes in adiposity measurements: a prespecified analysis of the SELECT trial. *The Lancet*, 2025; 406, 2257-2268). In contrast, the substantial subgroup of semaglutide patients losing more than 10% of baseline body weight had the lowest IR for MACE, even though a reduction MACE was seen in all semaglutide body weight change groups. Among placebo patients, the incidence of MACE tended to decrease with less body weight loss. Among semaglutide patients, the incidence of MACE tended to increase with less body weight loss (see also **Table S5 Figure S6**. This pattern may reflect the higher risks seen in patients experiencing unintended body weight loss due to other causes; and such patients may dominate the strata in the placebo group with higher body weight loss. Among semaglutide patients, a much higher proportion of patients lost more body weight, with IRs in those strata potentially dominated by patients benefiting from semaglutide's treatment effect on MACE observed in the trial. The same general patterns were observed when analyses were done in subgroups by sex.

In the categorization of SELECT patients by treatment arm and decrease in hsCRP by week 104, the lowest IR (1.68) occurred in those semaglutide-treated patients with the greatest hsCRP reduction (>50%, which included ~50% of all semaglutide-treated patients with events; associated HR of 0.63). In general, the patterns with body weight loss were less pronounced as compared to changes in hsCRP. The larger numbers of placebo patients in the strata with the greatest decrease in hsCRP may contribute to these results.

CV, cardiovascular; HR, hazard ratio; hsCRP, high-sensitivity C-reactive protein; IR, incidence rate; LOCF, last observation carried forward; MACE, major adverse cardiovascular events; MAR, missing at random; and MI, myocardial infarction.

Table S11. Linear continuous modeling of in-trial MACE risk by time-varying body weight loss or changes in hsCRP

Linear continuous modeling of MACE risk per 5% time-varying body weight loss (average of successive measurements)				
**All patients**				
**Semaglutide**		**Placebo**		**Interaction**
HR (95% CI); *P* value	Quadratic term *P* value	HR (95% CI); *P* value	Quadratic term *P* value	Semaglutide: Placebo HR (95% CI); *P* value
0.99 (0.93, 1.07); *P*=0.880	0.603	1.01 (0.99, 1.02); *P*=0.335	0.052	0.99 (0.97, 1.01); *P*=0.228
**Male patients**				
**Semaglutide**		**Placebo**		**Interaction**
HR (95% CI); *P* value	Quadratic term *P* value	HR (95% CI); *P* value	Quadratic term *P* value	Semaglutide: Placebo HR (95% CI); *P* value
0.99 (0.94, 1.05); *P*=0.793	0.225	1.01 (1.00, 1.03); *P*=0.072	0.121	0.99 (0.97, 1.01); *P*=0.320
**Female patients**				
**Semaglutide**		**Placebo**		**Interaction**
HR (95% CI); *P* value	Quadratic term *P* value	HR (95% CI); *P* value	Quadratic term *P* value	Semaglutide: Placebo HR (95% CI); *P* value
0.99 (0.91, 1.07); *P*=0.810	0.398	0.99 (0.97, 1.02); *P*=0.541	0.046	1.00 (0.97, 1.03); *P*=0.915

Linear continuous modeling of MACE risk per 25% time-varying decrease in hsCRP (average of successive measurements)				
**All patients**				
**Semaglutide**		**Placebo**		**Interaction**
HR (95% CI); *P* value	Quadratic term *P* value	HR (95% CI); *P* value	Quadratic term *P* value	Semaglutide: Placebo HR (95% CI); *P* value
0.93 (0.91, 0.96); *P*<0.0001	0.149	0.92 (0.89, 0.94); *P*<0.0001	0.390	1.02 (0.99, 1.06); *P*=0.217
**Male patients**				
**Semaglutide**		**Placebo**		**Interaction**
HR (95% CI); *P* value	Quadratic term *P* value	HR (95% CI); *P* value	Quadratic term *P* value	Semaglutide: Placebo HR (95% CI); *P* value
0.93 (0.90, 0.96); *P*<0.0001	0.166	0.90 (0.87, 0.93); *P*<0.0001	0.149	1.04 (1.00, 1.08); *P*=0.055
**Female patients**				
**Semaglutide**		**Placebo**		**Interaction**
HR (95% CI); *P* value	Quadratic term *P* value	HR (95% CI); *P* value	Quadratic term *P* value	Semaglutide: Placebo HR (95% CI); *P* value
0.93 (0.87, 0.99); *P*=0.016	0.424	1.00 (0.93, 1.07); *P*=0.907	0.679	0.95 (0.87, 1.03); *P*=0.203

Associations between risk of MACE and time-varying measures of change in body weight or hsCRP follow the same patterns as seen with associations with measurements at week 104.

HR, hazard ratio; hsCRP, high-sensitivity C-reactive protein; and MACE, major adverse cardiovascular events.

SUPPLEMENTAL FIGURES

CIRCULATIONAHA/2025/074482, Plutzky, et al.
"Effect of semaglutide on the inflammatory biomarker high-sensitivity C-reactive protein in people with established cardiovascular disease and overweight or obesity in SELECT: A prespecified secondary analysis"

Index

Supplemental Figures:

Figure S1. hsCRP and body weight percentage change from baseline to week 208

Figure S2. Absolute change in hsCRP over time to week 104

Figure S3. Forest plot of ratio-to-baseline hsCRP at week 104 by baseline hsCRP subgroup and treatment arm

Figure S4. Survival curves of time to selected CV outcomes by baseline hsCRP tertiles

Figure S5. Ratio-to-baseline hsCRP by change in observed body weight reduction (<5, 5–<10, 10–<15, ≥15%) among patients at week 104 from the first on-treatment observation period

Figure S6. Estimated MACE incidence rates by percent change in body weight or change in log hsCRP at week 104

Figure S1. hsCRP and body weight percentage change from baseline to week 208

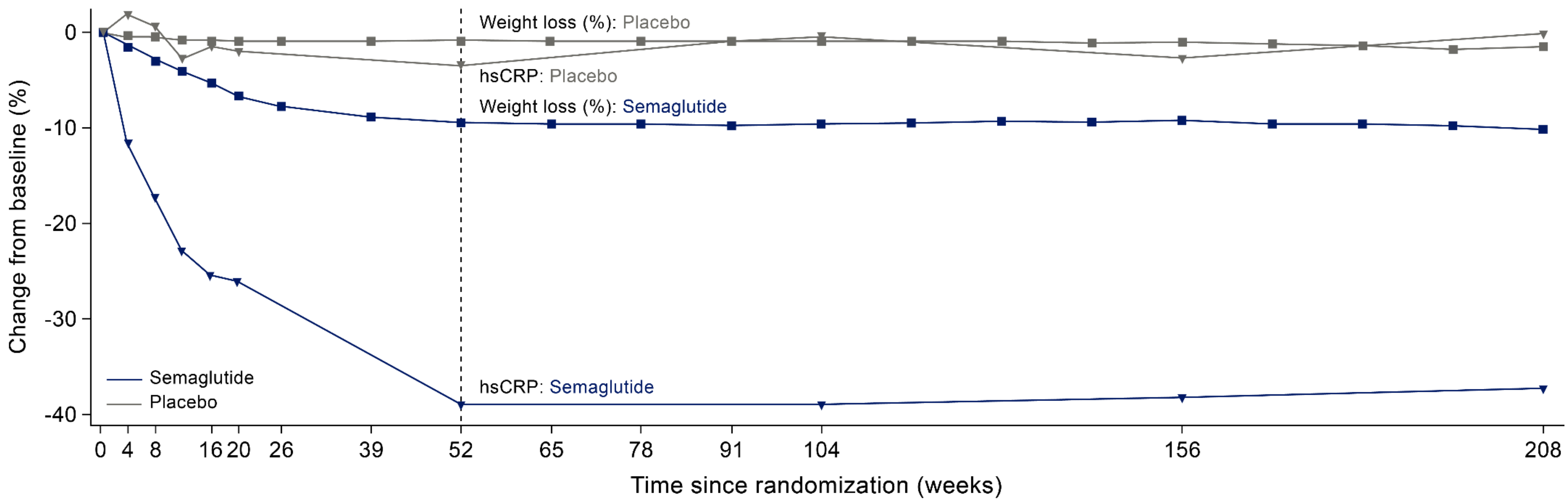

Data are mean percent change from baseline in body weight and geometric mean percent change from baseline in hsCRP, analyzed using a mixed model for repeated measurements with treatment group as a fixed factor and baseline value as a covariate, all nested within visit, based on in-trial data. The model was fitted to all patients with a measurement, regardless of treatment status at week 208. Mean estimates were adjusted according to observed baseline distribution. Week 4–16 measurements are for 6011 randomized patients in whom extra laboratory measurements were mandated by local safety guidance (Austria, Belgium, the Czech Republic, Denmark, Finland, France, Germany, Greece, Hungary, Ireland, Italy, Latvia, the Netherlands, Norway, Poland, Portugal, Romania, Spain, Sweden, and the United Kingdom). The basis for showing percent changes in hsCRP and body weight together is supported by the similar change in these measures; in linear continuous modeling among placebo patients (**Table S5**), each 25% lower hsCRP at baseline was associated with 6% lower risk of MACE (HR 0.94; 95% CI 0.93, 0.96), while each 5 kg lower body weight at baseline (approximately 5.1% of the mean body weight of 96.8 kg) was associated with 1% lower risk of MACE (HR 0.99; 95% CI 0.97, 1.01).

HR, hazard ratio; hsCRP, high-sensitivity C-reactive protein; MACE, major adverse cardiovascular event.

Figure S2. Absolute change in hsCRP over time to week 104

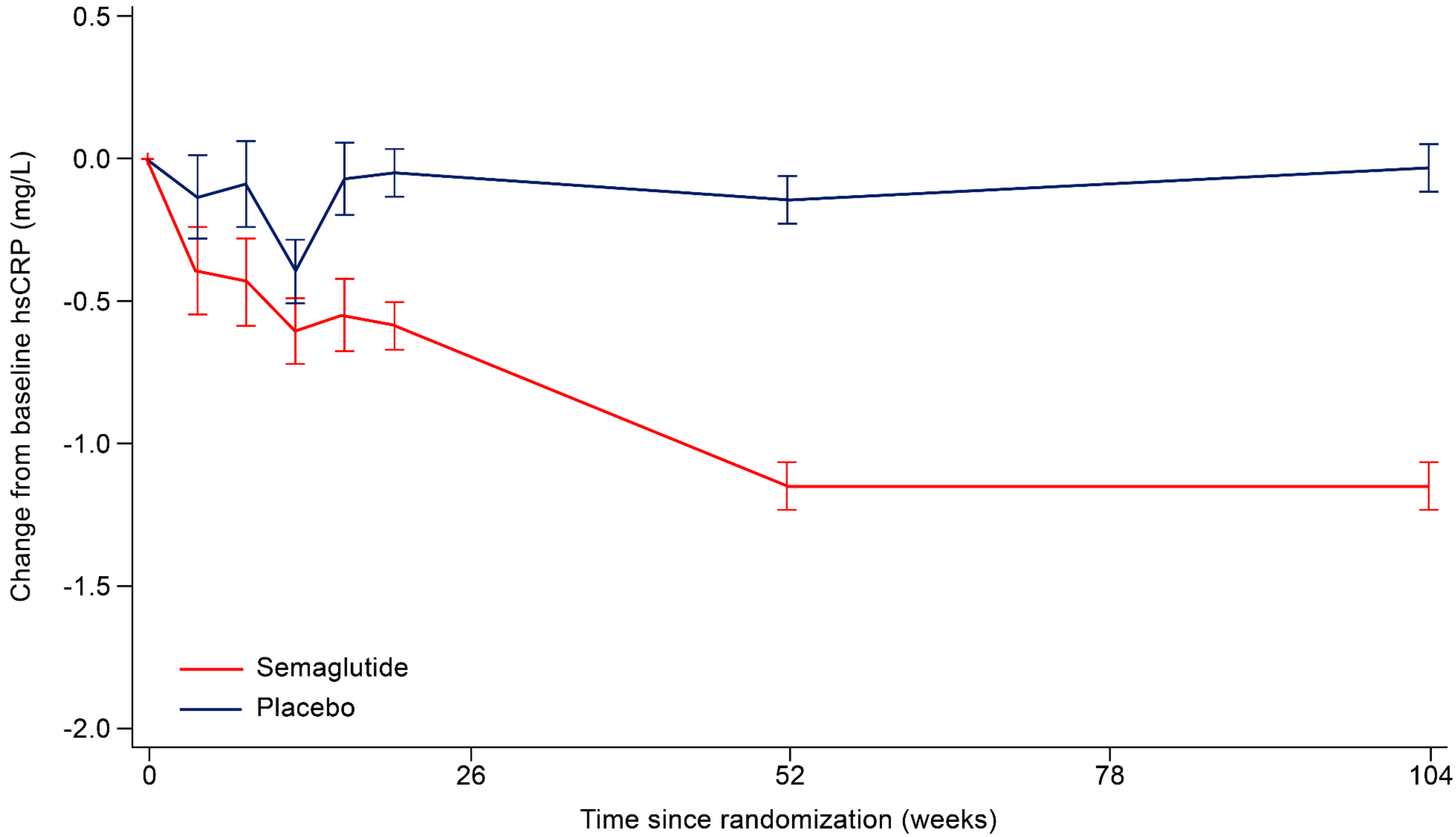

Data are estimated mean (SE) changes from baseline, analyzed using a mixed model for repeated measurements with treatment group as a fixed factor and baseline value as a covariate, all nested within visit, based on in-trial observation period data. The model was fitted to all patients with a measurement, regardless of treatment status at week 104. Mean estimates were adjusted according to observed baseline distribution. Week 4–16 measurements are for 6011 randomized patients with mandated extra laboratory measurements (see **Figure S1**).

hsCRP, high-sensitivity C-reactive protein.

Figure S3. Forest plot of ratio-to-baseline hsCRP at week 104 by baseline hsCRP subgroup and treatment arm

	Ratio to baseline in hsCRP			
	**Semaglutide**	**Placebo**	**ETR (95% CI)**	**Interaction *P* value**
**Baseline hsCRP subgroup**				0.8001
<1 mg/L	0.52 (n=2483)	0.82 (n=2552)	0.63 (0.59, 0.66)	
1–<2 mg/L	0.60 (n=2026)	0.98 (n=2036)	0.61 (0.57, 0.65)	
2–<5 mg/L	0.67 (n=2324)	1.09 (n=2292)	0.62 (0.58, 0.65)	
5–<10 mg/L	0.75 (n=1041)	1.15 (n=1062)	0.65 (0.60, 0.71)	
≥10 mg/L	0.63 (n=663)	1.01 (n=584)	0.62 (0.56, 0.70)	

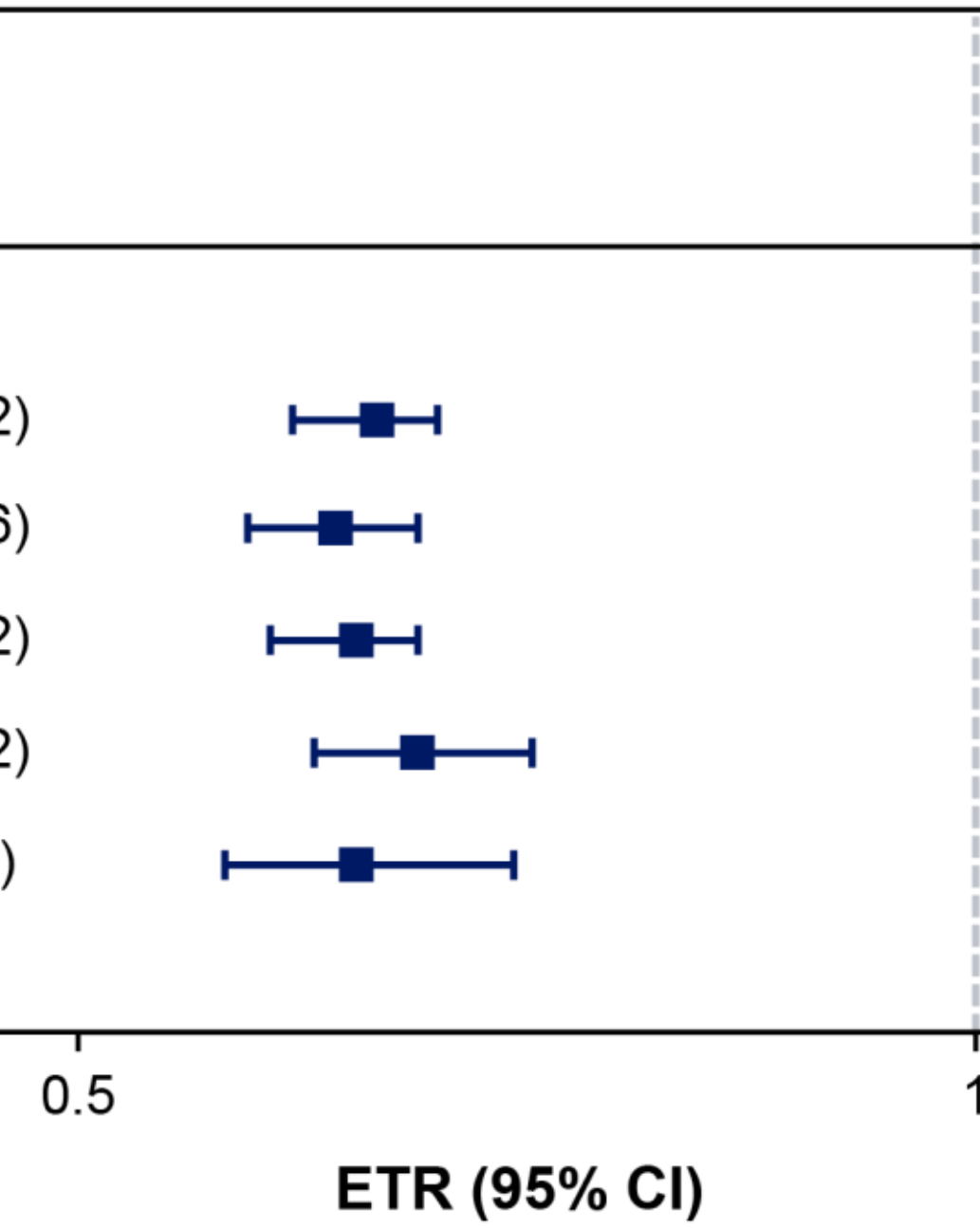

Data are estimated geometric mean ratios to baseline and ETRs, analyzed using an analysis of covariance with treatment group, subgroup, and treatment group-by-subgroup interaction as fixed factors, and baseline value as a covariate (in-trial observation period data). Multiple imputation via linear regression was used to impute missing week 104 data separately for each treatment group. The model was fitted to all patients with a measurement regardless of treatment status at week 104. Mean estimates were adjusted according to observed baseline distribution. hsCRP observations were log-transformed prior to analysis and the estimates back-transformed afterwards. (n) denotes the number of patients contributing to the analysis.

ETR, estimated treatment ratio; and hsCRP, high-sensitivity C-reactive protein.

Figure S4. Survival curves of time to selected CV outcomes by baseline hsCRP tertiles

A) MACE

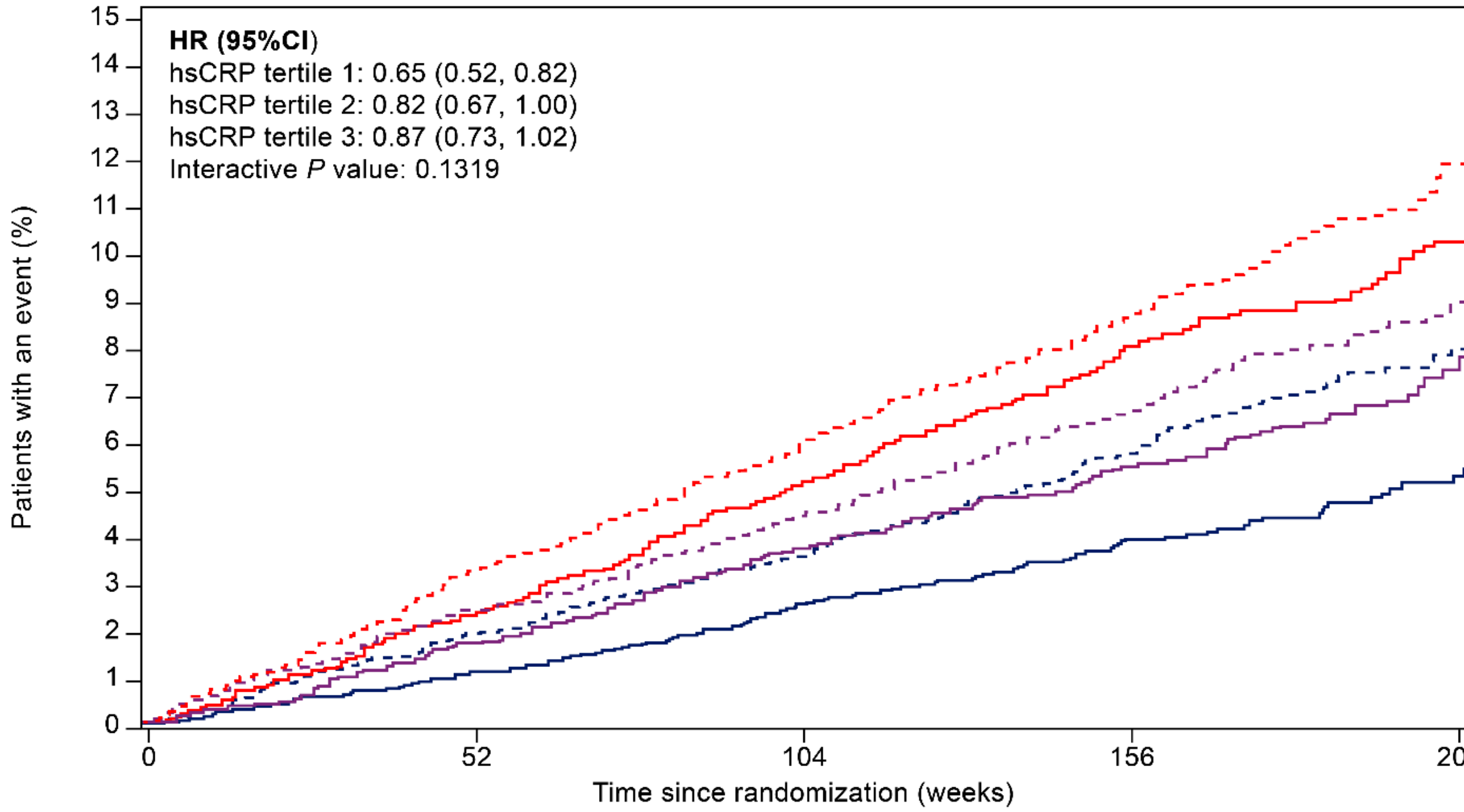

B) Death from CV causes

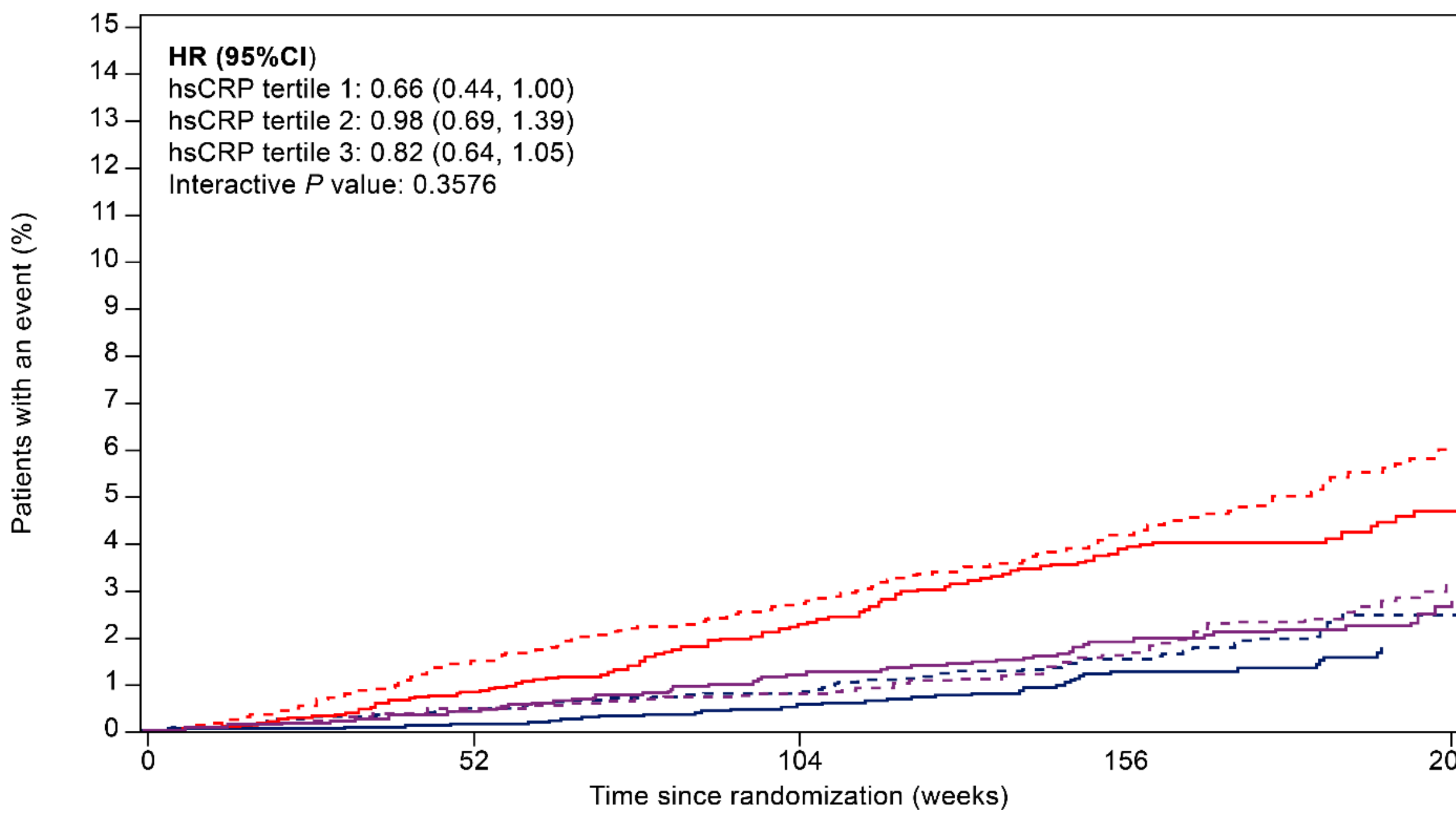

C) All-cause death

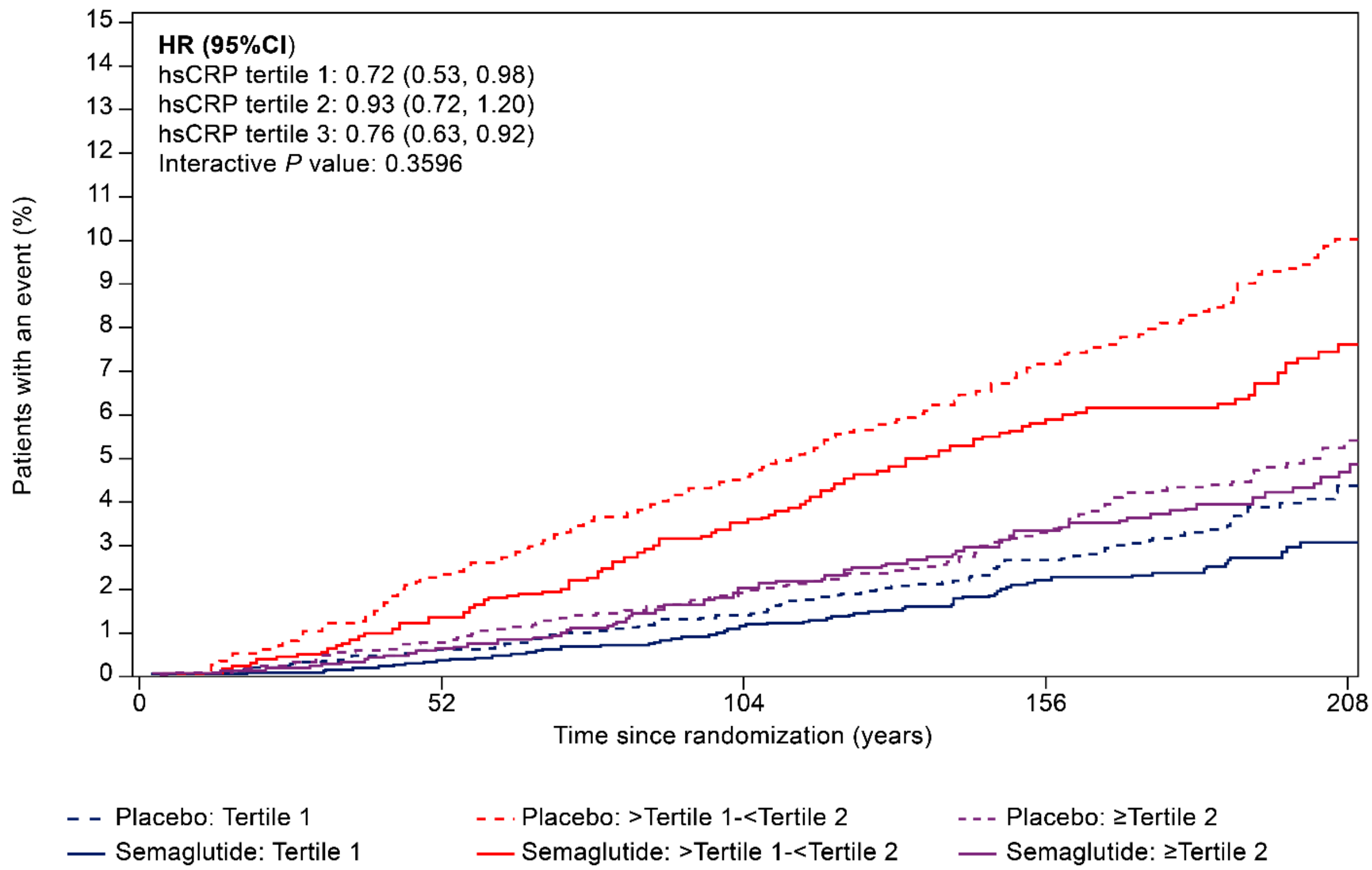

Data are the observed, ie, as measured, cumulative incidence rates of patients experiencing their first occurrence of each CV outcome shown, calculated using the Aalen–Johansen method, with all non–CV-related death as a competing risk, and the estimated HRs, analyzed using a Cox proportional hazards model with treatment group, subgroup, and treatment group-by-subgroup interactions as fixed factors (in-trial observation period data).

CV, cardiovascular; HR, hazard ratio; hsCRP, high-sensitivity C-reactive protein; MACE, major adverse cardiovascular event and Sema, semaglutide.

Figure S5. Ratio-to-baseline hsCRP by change in observed body weight reduction (<5, 5–<10, 10–<15, ≥15%) among patients at week 104 from the first on-treatment observation period

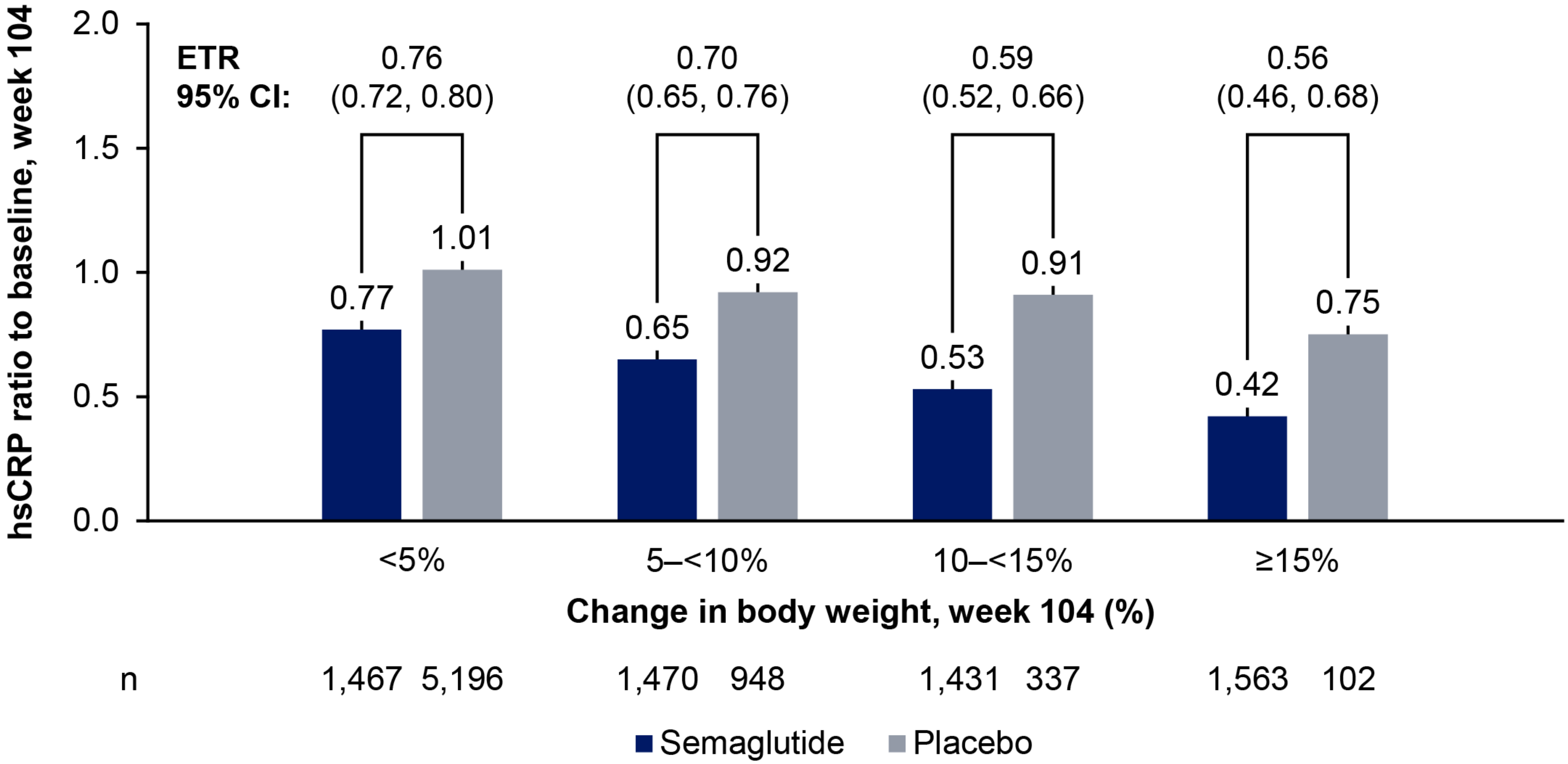

Data are estimated geometric mean ratios to baseline and ETRs, analyzed using an analysis of covariance with treatment group, subgroup, and treatment group-by-subgroup interaction as fixed factors, and baseline body weight and hsCRP as covariates, using observed (ie, as-measured) data from the first on-treatment observation period. Baseline hsCRP was log-transformed prior to analysis and the estimates back-transformed afterwards. n denotes the number of patients contributing to the analysis. Interaction *P* value: <0.001. Linear trend test *P* value: 0.0004.

ETR, estimated treatment ratio; and hsCRP, high-sensitivity C-reactive protein.

Figure S6. Estimated MACE incidence rates by percent change in body weight or change in log hsCRP at week 104

A) MACE incidence rates by percent change in body weight from baseline to week 104

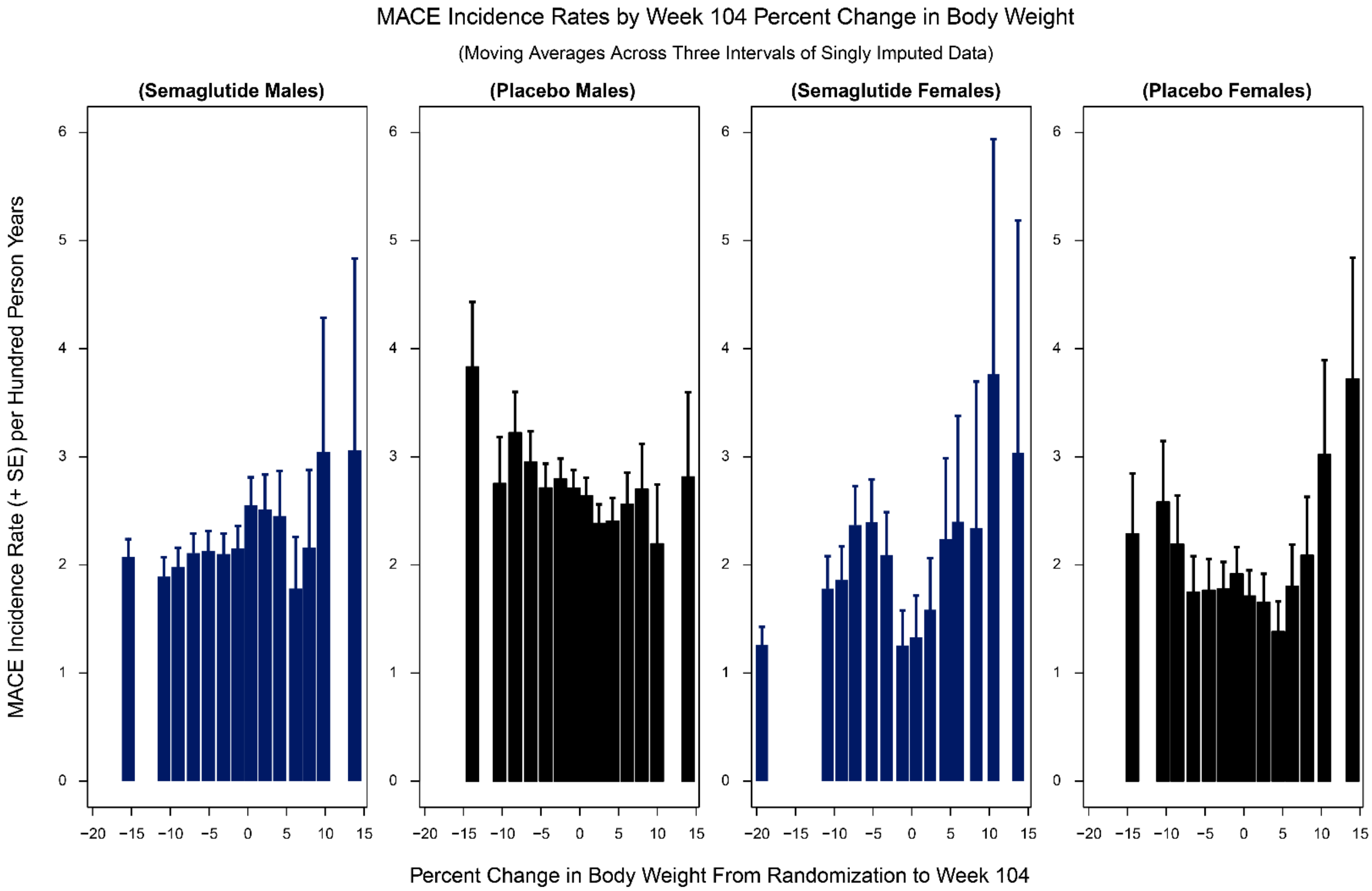

B) MACE incidence rates by change in log hsCRP (week 104) to baseline

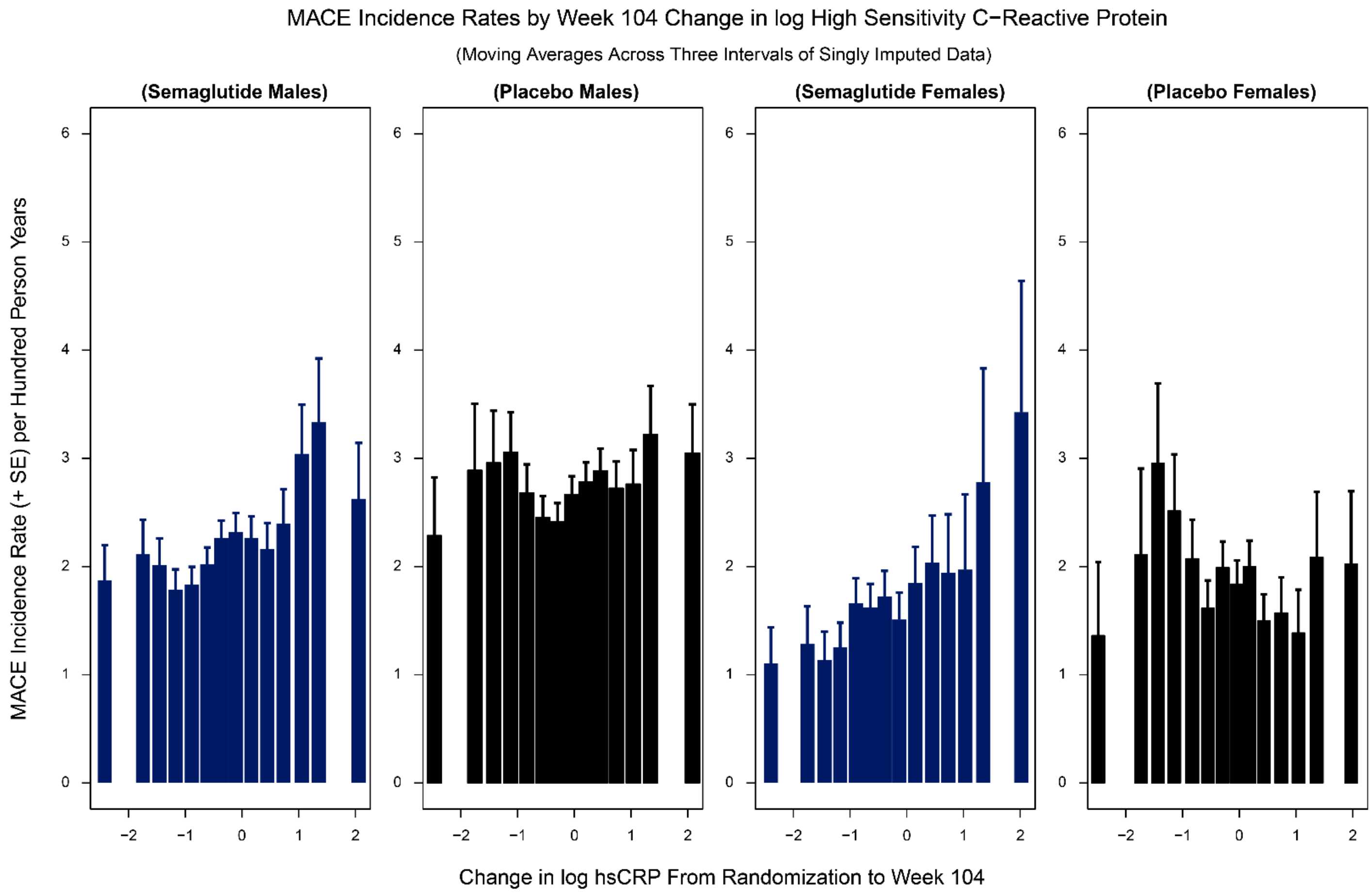

Graphical description (smoothed moving average) of MACE incidence rates with SEs by **A**, Percent change in body weight from baseline to week 104; **B**, Log ratio of hsCRP at week 104 to baseline, shown separately for each sex and treatment arm. For both markers of weight and hsCRP, changes between week 104 and baseline were divided into the same evenly spaced categories for all subgroups. Any missing data prior to an observed death was singly imputed using a missing at random model based on all available measurements for each patient with last observation carried forward for any subject observed to die before week 104 (see Supplemental Methods Overview). In the moving average, incidence rates and SEs were computed for every combination of three adjacent intervals and plotted against the average marker value within those three intervals.

Discussion: To provide finer descriptive detail about any possible nonlinearity in the associations between risk of MACE and changes in weight or hsCRP by week 104, **Figure S1** presents a smoothed moving average of incidence rates according to changes in these markers for each treatment arm and sex subgroup. Of note, these plots do not adjust for associations between baseline measurements (as done in **Table S9**) nor the change in measurements (as done in **Table S6**) nor for the associations between risk of MACE and baseline weight or hsCRP (as done in **Table S7**). Also of note, female patients account for 27.7% of the total SELECT sample but only 21.4% of events; the lower precision of estimates among female patients is evidenced by the larger standard error bars.
Although a significant reduction in risk of MACE occurred in the semaglutide group versus placebo in SELECT, general relative trends are seen here in the semaglutide arm in which lesser weight loss and lesser decreases in hsCRP were associated with less MACE benefit, as evident in both male and female patients. In contrast and for comparison, as reported, in the placebo arm, a paradoxical trend is seen with the greatest weight loss being associated with a higher risk of MACE, as more apparent in male than in female patients.

Among placebo patients, any consistent trend toward change in hsCRP being prognostic for risk of MACE is much less evident; explanations for the pattern seen in female patients remains unclear.
The disparities seen between treatment arms in the prognostic value of changes in weight and hsCRP on risk of MACE are consistent with neither of these markers alone being able to completely explain semaglutide's effects on MACE, as also noted in the manuscript.

hsCRP, high-sensitivity C-reactive protein; MACE, major adverse cardiovascular event.
